# APOA2 increases cholesterol efflux capacity to plasma HDL by displacing the C-terminus of resident APOA1

**DOI:** 10.1016/j.jlr.2024.100686

**Published:** 2024-10-28

**Authors:** Snigdha Sarkar, Jamie Morris, Youngki You, Hannah Sexmith, Scott E. Street, Stephanie M. Thibert, Isaac K. Attah, Chelsea M. Hutchinson Bunch, Irina V. Novikova, James E. Evans, Amy S. Shah, Scott M. Gordon, Jere P. Segrest, Karin E. Bornfeldt, Tomas Vaisar, Jay W. Heinecke, W. Sean Davidson, John T. Melchior

**Affiliations:** 1 Biological Sciences Division, Pacific Northwest National Laboratory, Richland, WA, USA; 2 Department of Pathology and Laboratory Medicine, University of Cincinnati, Cincinnati, OH, USA; 3 Department of Pediatrics, Cincinnati Children’s Hospital Medical Center and the University of Cincinnati, Cincinnati, OH, USA; 4 Environmental Molecular Sciences Laboratory, Pacific Northwest National Laboratory, Richland, WA, USA; 5 Department of Physiology and the Saha Cardiovascular Research Center, University of Kentucky College of Medicine, Lexington, KY, USA; 6 Department of Medicine, Vanderbilt University Medical Center, Nashville, TN, USA; 7 Department of Medicine, UW Medicine Diabetes Institute, University of Washington, Seattle WA, USA; 8 Department of Neurology, Oregon Health and Science University, Portland, OR, USA

**Keywords:** Apolipoprotein(s), Apolipoprotein A-I, Apolipoprotein A-II, ABCA1, Cholesterol Efflux, High Density Lipoproteins, Lipoprotein metabolism, Lipoproteins, Structure

## Abstract

The ability of high-density lipoprotein (HDL) to promote cellular cholesterol efflux is a more robust predictor of cardiovascular disease protection than HDL-cholesterol levels in plasma. Previously, we found that lipidated HDL containing both apolipoprotein A-I (APOA1) and A-II (APOA2) promotes cholesterol efflux via the ATP-binding cassette transporter (ABCA1). In the current study, we directly added purified, lipid-free APOA2 to human plasma and found a dose-dependent increase in whole plasma cholesterol efflux capacity. APOA2 likewise increased the cholesterol efflux capacity of isolated HDL with the maximum effect occurring when equal masses of APOA1 and APOA2 coexisted on the particles. Follow-up experiments with reconstituted HDL corroborated that the presence of both APOA1 and APOA2 were necessary for the increased efflux. Using limited proteolysis and chemical cross-linking mass spectrometry, we found that APOA2 induced a conformational change in the N- and C-terminal helices of APOA1. Using reconstituted HDL with APOA1 deletion mutants, we further showed that APOA2 lost its ability to stimulate ABCA1 efflux to HDL if the C-terminal domain of APOA1 was absent, but retained this ability when the N-terminal domain was absent. Based on these findings, we propose a model in which APOA2 displaces the C-terminal helix of APOA1 from the HDL surface which can then interact with ABCA1—much like it does in lipid-poor APOA1. These findings suggest APOA2 may be a novel therapeutic target given this ability to open a large, high-capacity pool of HDL particles to enhance ABCA1-mediated cholesterol efflux.

Atherosclerosis, a leading cause of coronary heart disease (CHD), is linked to an imbalance in cholesterol deposition and removal from artery walls (1, 2). A key pathway for cholesterol removal is reverse cholesterol transport, where high-density lipoproteins (HDL) in plasma accept phospholipid and cholesterol from macrophages and other arterial cells for return to the liver (2). Research shows that HDL's ability to accept and transport excess lipid is a better marker for CHD than traditional HDL particle cholesterol content (HDL-C) (3, 4). Thus, identifying HDL features that enhance cholesterol efflux capacity (CEC) is crucial for potential CHD therapies.

HDL promotes cellular cholesterol removal through both “passive” and “active” mechanisms, with apolipoprotein A-I (APOA1) acting as the primary cholesterol acceptor (5, 6). Passive cholesterol efflux occurs via aqueous diffusion, transferring cholesterol from cells to HDL down a concentration gradient. This is facilitated by cell surface proteins like SR-B1 and is associated with lipid efflux to lipid-containing, mature HDL particles (6). In contrast, the active mechanism is driven by ATP-binding cassette transporter (ABCA1) and is mostly triggered by lipid-poor apolipoproteins—primarily APOA1—and is critical for HDL biogenesis (7).

Apolipoprotein A-II (APOA2), the second most abundant protein in human HDL, is a 17.4 kDa homodimer linked by a disulfide bond (8). Its role in lipoprotein metabolism is debated, with studies showing both pro- and anti-atherogenic effects in mouse models (9, 10). Its specific role in cholesterol efflux to HDL remains unclear. In 1984, Cheung and Albers characterized human plasma HDLs with and without APOA2, finding that "LpA-I" particles contained APOA1 but no APOA2, while "LpA-I/A-II" contained both proteins (11). Recent shotgun proteomics revealed that while many proteins were shared, LpA-I had unique proteins linked to inflammatory response and immunity, whereas LpA-I/A-II proteins were primarily associated with lipid transport (12, 13). Notably, we found that LpA-I/A-II particles outperformed LpA-I in accepting cellular cholesterol, especially under conditions where ABCA1 expression was induced by cAMP (13, 14).

Here, we investigated the mechanistic basis behind APOA2’s influence on cholesterol efflux to HDL. We hypothesized that APOA2 modifies HDL structure to enhance ABCA1-mediated cholesterol efflux. We evaluated this in complex systems like human plasma and in isolated HDL by adding exogenous APOA2 and measuring its impact on ABCA1-mediated cholesterol efflux. After confirming that physiological levels of APOA2 significantly alter HDL CEC, we used mass spectrometry-based structural biology, selective particle reconstitution, and deletion mutagenesis to propose a mechanism for APOA2's enhancement of ABCA1-mediated cholesterol efflux to mature HDL particles. We also discuss the potential therapeutic implications of our findings.

## Materials and methods

### General materials

Proteinase K (Tritirachium album) was purchased from Millipore Sigma. Bis-sulfosuccinimidyl-suberate (BS3-H_12_/D_12_) was purchased from Creative Molecules Inc. Disuccinimidyl dibutyric urea (DSBU) and disuccinimidyl sulfoxide (DSSO) were purchased from Thermo Fisher Scientific. For protein quantification, BCA protein assay kit (Micro BCA™ protein assay kit, Pierce) was used. Human plasma was obtained through the Hoxworth Blood Bank from one normolipidemic, human male volunteer.

### Isolation of HDL from human plasma

To isolate plasma HDL using sequential ultracentrifugation (UC-HDL or UC-isolated HDL), the density of fresh plasma was adjusted to 1.006 g/ml by the addition of potassium bromide. The sample was centrifuged at 360,000 × g and 5°C for 24 h to isolate and remove VLDL and chylomicrons in the top fraction. The density of the bottom fraction was adjusted to 1.063 g/ml and centrifuged at 360,000 × g and 5°C for 24 h to isolate and remove LDL in the top fraction. The density of the bottom fraction was adjusted to 1.21 g/ml and centrifuged at 360,000 × g and 5°C for 48 h. The top fraction containing the purified HDLs was collected and dialyzed into PBS (10 mM PBS, 140 mM NaCl, 0.01% EDTA, 0.01% azide, pH 7.4) (15).

### Isolation of human plasma APOA2

Lipid-free plasma APOA2 was isolated from human plasma obtained from Hoxworth Blood Bank. First, HDL was isolated using sequential density ultracentrifugation as described. UC-isolated HDL was then delipidated using a modified Folch extraction method (16) and dialyzed against 10 mM ammonium bicarbonate, lyophilized, and stored at −80°C until ready for further purification. Next, plasma APOA2 was then purified from delipidated HDL protein by fast protein liquid chromatography with an anion exchange chromatography column (HiPrep Q Sepharose Fast Flow 16/10 column, Cytiva Cat # 28936543). Briefly, 50–100 mg HDL protein was solubilized and denatured in 6 M Urea, 20 mM Tris HCl, pH 7.4 (start buffer) overnight at 4°C. HDL proteins were then purified using a step gradient of 10%–30% NaCl elution buffer (6M Urea, 20 mM Tris HCl, 200 mM NaCl, pH 7.4). Pure, lipid-free plasma APOA2 protein was dialyzed into 10 mM ammonium bicarbonate, lyophilized, and stored at −80°C until ready for further use.

### Exogenous addition of APOA2 to plasma HDL

Lyophilized APOA2 was solubilized in 3 M guanidine hydrochloride and incubated overnight on a rotating rack at 4°C to ensure complete protein unfolding. The protein was refolded by dialyzing into STB (10 mM Tris HCl, 150 mM NaCl, 1 mM EDTA, 0.02% NaN3, pH 8.2) and the final concentration was determined using a Markwell Lowry assay (17). For the respective incubation experiments with plasma, APOB-depleted plasma, and UC-isolated HDL, lipid-free APOA2 was added as a mass percentage of APOA1 content in the sample which was estimated as 78% of the total HDL protein concentration. Following the addition, the sample and APOA2 were incubated at 37°C for 1 h and immediately profiled using size-exclusion chromatography (SEC). Plasma samples enriched with APOA2 were subjected to SEC using two Superose 6 columns connected in tandem after APOA2 incubation. For studies using UC-isolated HDL, the incubated samples were applied to two Superdex 200 Increase size-exclusion columns instead, to separate the HDL-bound and lipid-free proteins. The fractions containing HDL-bound particles and fractions containing displaced lipid-free proteins were pooled separately. Before processing the samples for mass spectrometry, the pooled fractions were concentrated using Amicon Ultra 3 kDa centrifugal concentrators following vendor protocol.

### Depletion of APOB-containing lipoproteins from plasma

APOB-containing particles were removed from plasma as previously described (18). Briefly, 1 ml of plasma was mixed with 400 μl of 20% PEG (MW 6000, Sigma Aldrich; St. Louis, MO) and incubated at room temperature for 20 min. The mixture was centrifuged for 30 min at 10,000 rpm to pellet precipitated APOB-containing lipoproteins bound to the PEG. Supernatant containing non-APOB particles (or HDL) was collected for subsequent assays.

### Synthesis of reconstituted HDL particles with POPC

Three different types of reconstituted HDL were generated: recombinant HDLs (rHDLs) that contain only APOA1, rHDLs that contain only APOA2, and rHDLs that contain both APOA1 and APOA2. In all cases, lyophilized protein (APOA1, APOA2) was solubilized and denatured in STB (10 mM Tris/HCl, 1 mM EDTA, 0.2% NaN3,150 mM NaCl) containing 3 M guanidine HCl for 1 h at 4°C. This was followed by refolding at 4°C by dialyzing against three changes of 4L STB for a minimum of 3 h each. All particles were synthesized by combining the protein with 1-palmitoyl-2-oleoyl-sn-glycero-3-phosphatidylcholine (POPC, Avanti Polar Lipids) at ratios that generated homogenous, size-matched particles. For APOA1-only rHDL, particles were generated using a molar ratio 85:1 POPC:APOA1. For the APOA2-only rHDL, particles were generated using a molar ratio of 58:1 POPC:APOA2 assuming APOA2 exists as a dimer. All particles were generated with a modified sodium cholate dialysis method as previously described using deoxycholic acid sodium salt (Fisher Scientific) (19) to first generate detergent micelles with the POPC. Protein was added at the molar ratios specified, particles were incubated for 1 h at 37°C, and the deoxycholic acid was removed through sequential dialysis. For the rHDL that contained both APOA1 and APOA2, we first generated APOA1-only rHDL as described above. Particles were diluted to 0.1 mg/ml and allowed to equilibrate for 24 h at 4°C. Lipid-free APOA2 was reduced with 3 mM DTT and added to the APOA1 rHDL at a molar ratio of 0.475:1 APOA2:APOA1 to avoid displacement of APOA1. Particles were incubated for 48 h at 4°C and purified by SEC using three superdex 200 10/300 columns in series. Particle concentrations were determined by quantifying protein using a modified Markwell-Lowry assay. Particle size and homogeneity were confirmed by 8%–25% nondenaturing gradient gel electrophoresis (NDGGE) (GE Healthcare Life Sciences) with Coomassie blue staining. Presence of proteins were confirmed using denaturing SDS 4%–15% gel (Mini-PROTEAN TGX, BioRad) with Coomassie blue staining.

### Limited proteolysis on HDL

The UC-HDL particles enriched with exogenous APOA2 were split into two identical sample subsets for limited proteolysis (LiP) experiments (20, 21). The first set (LiP samples) was treated with a nonspecific protease (Proteinase K) at an enzyme-substrate ratio of 1:200 (w/w) for 1 min at 25°C. The reaction was quenched by heating the samples for 5 min at 98°C. The samples were denatured in 8 M urea and prepared for mass spectrometry analysis. The second set (global proteomics samples) served as control samples, which were treated identically but with buffer was added in the place of Proteinase K. The displaced lipid-free proteins were directly denatured with 8M urea. After denaturation, proteins in all samples were reduced by incubating the samples with 5 mM DTT for 1 h at 25°C and cysteines were alkylated by incubating samples in 40 mM iodoacetamide at 25°C for 45 min in dark. The samples were diluted 3-fold with 50 mM ammonium bicarbonate. Subsequently, samples were digested twice, first with LysC at an enzyme:substrate ratio of 1:100 (w/w) for 2 h at 25°C, shaking at 850 rpm, followed by digestion with sequencing grade trypsin with an enzyme:substrate ratio of 1:100 (w/w) for 16 h at 25°C, shaking at 850 rpm. The protease activity was quenched by the addition of 0.1% TFA. Digested peptides were applied to C18 96-well plate (Phenomenex Part No. 8E-S001-BGB), desalted, and eluted using 80% acetonitrile, 0.1% TFA. The eluted peptides were dried and stored at −20°C until ready for LC-MS/MS analysis.

### Cross-linking and sample preparation for mass spectrometry

Samples were cross-linked with a variety of reagents including BS3-H_12_/D_12_, DSBU, and DSSO. Cross-linking was performed using a final concentration of 0.8, 2.5 mM, and 1.5 mM for the BS3, DBSU, and DSSO, respectively. Samples were cross-linked at a final protein concentration between 0.5 – 1.0 mg/ml. BS3 cross-linking was performed in PBS at 4°C for 2 h. DSBU cross-linking was performed in 22.5 mM Hepes (pH 7.5), 67.5 mM NaCl for 1 h at room temperature. DSSO cross-linking was also performed in Hepes buffer for 1 h at room temperature. All samples were quenched by the addition of at least a 10-fold molar excess of Tris buffer. Samples were subsequently denatured, digested, and prepared for mass spectrometry analysis as described above.

### Data acquisition using mass spectrometry

Global proteomics and LiP samples were analyzed using a Q-Exactive HF-X mass spectrometer (Thermo Fisher Scientific). Peptides were reconstituted in milli-Q water and separated using a Thermo Dionex Ultimate 3000 liquid chromatography (LC) and C18 reverse phase column (30 cm × 75 μm i.d., 1.7 μm particle size of Waters Acquity BEH particles, Waters) prepped in-house. The LC was configured with 2 pumps, one pump was used for sample trapping and the other for reverse-flow elution onto the C18 column. The trap column consisted of an in-house prepared 4-cm × 100 um i.d. packed with 5-μm Jupiter C2. After trapping, peptides were introduced onto the C18 column at 200 nl/min with buffer A (0.1% formic acid in H2O) and buffer B (0.1% formic acid in acetonitrile). Peptides were eluted over 60 min using the following gradients (time in minutes, %B): 1) 2–6 min, 1%–8% B; 2) 6–54 min, 8%–25% B; 3) 54–59 min, 25%–35% B. MS1 spectra were acquired from 380 to 1800 m/z at a resolution of 60 k. The top 12 FT-HCD-MS/MS spectra were acquired in data-dependent mode using an isolation window of 0.7 m/z at a resolution of 45 k. For HCD fragmentation, a normalized collision energy of 30 was used with a 45 s exclusion time, analyzing charge states 2 to 6.

Cross-linked peptides from BS3 and DSBU cross-linking studies were analyzed using an Orbitrap Fusion Lumos mass spectrometer (Thermo Fisher Scientific). Peptides were reconstituted in 0.1% formic acid and separated using a nanoACQUITY UPLC system (Waters) and C18 reverse phase column (20 cm × 75 μm i.d., 1.9 μm particle size, ReproSil-Pur 120 C18-AQ, Dr Maisch GmbH) prepared in-house. Peptides were introduced onto the C18 column operating at 200 nl/min with buffer A (0.1% formic acid in H_2_O) and buffer B (0.1% formic acid in acetonitrile). Peptides were eluted over 190-min using the following gradients (time in minutes, %B): 1) 30–31 min, 2%–4% B; 2) 31–115 min, 4%–30% B; 3) 115–124 min, 30%–60% BMS1 spectra was acquired from 400-2000 m/z at a resolution of 120 k. MS/MS spectra were acquired in data-dependent mode using an isolation window of 2.0 m/z at a resolution of 30 k. Peptides were fragmented using stepped HCD of 20, 30, 40% collision energy with a 45 s exclusion time, analyzing charge states +3 to +10.

For DSSO samples, high pH reversed-phase LC was performed on cross-linked and digested HDL peptides using an offline Agilent 1200 series HPLC. Fifteen concatenated fractions were analyzed by Orbitrap Lumos Fusion Tribrid mass spectrometer (Thermo Fisher Scientific, Waltham, MA), using a hybrid CID-MS/MS and CID-MS3 fragmentation scheme, as previously described (22).

### Proteomic data analysis

#### Protein abundance

Raw files (.raw) were searched against a Homo Sapiens Uniprot/SwissProt database (26, 813 entries) using MaxQuant (Ver. 1.6.17.0) for label-free quantification (LFQ). Protein abundance changes were determined on samples only treated with trypsin (global proteomics samples) with a maximum of four missed cleavages. Searches included variable modifications of N-terminal peptide acetylation and oxidation at methionine and the fixed modification of carbamidomethylation. False discovery rates (FDR) of proteins and peptides was set to < 1%. All searches included match between runs with a window of 0.7 min. Identified proteins were limited to those with at least two unique peptides. Data was further processed with the R package RomicsProcessor (ver 1.1.0,https://github.com/PNNL-Comp-Mass-Spec/RomicsProcessor). Briefly, LFQ intensities from MaxQuant were log2 transformed and filtered to only include proteins with a maximum missingness of 50% within one experimental group. The total LFQ intensities were normalized by the median to correct for subtle changes in mass amounts introduced into the mass spectrometer. Missing values were imputed using the method developed by Tyranova *et al*. to calculate statistical parameters (23). A two-sample *t* test was performed to identify significantly altered proteins between experimental and control groups (*P* < 0.05).

#### Protein structure

Structural changes were determined on samples treated with both proteinase K and trypsin (LiP samples). In this case, searches were performed as above but with additional cleavage sites at alanine, glycine, isoleucine, leucine, methionine, proline, valine, phenylalanine, tryptophan, and tyrosine which we manually added into MaxQuant. The maximum missed cleavage was set to 20 to account for all the additional cleavage sites. For identifying peptide changes within the limited proteolysis experiments, individual peptide fold-changes were corrected if the related protein was found to be statistically significant in the samples treated with trypsin alone. To correct the peptide-level fold changes in the LiP samples, the protein-level fold changes determined in the global proteomics samples were used. This was done to identify significant peptide changes caused by protein structural alteration alone and not due to changes in the number of protein copies. A two-sample *t* test was performed to identify statistically different peptides across groups (*P* < 0.01).

Structural changes are visualized in the form of 2D-structural barcode. To create the structural barcodes, the residue-level fold changes of statistically significant semi-tryptic peptides (*P*-value <0.01) are represented in the form of a heatmap. Fold changes were calculated by comparing experimental groups with exogenous APOA2 to the control group with 0% exogenous APOA2. Regions with net positive fold change are shown in red, while those with net negative fold change are shown in blue. The fold changes for each peptide in the double-digested samples were corrected by the statistically significant APOA1 protein fold change from the trypsin-only sample by dividing the peptide-level fold change with the protein-level fold change. This was done to nullify the effect of the abundance changes from the structural data. The fold change of statistically significant semi-tryptic peptides was equally distributed across the encompassing amino acid. The residue-level fold changes resulting from different peptides were summed to create the structural barcodes which are aligned along the APOA1 amino acid sequence.

#### Cross-linking

Peptides cross-linked with BS3-H_12_D_12_ were identified from the .raw files using Spectrum Identification Machine for Cross-linked Peptides (SIM-XL, version 1.5.7.2), as previously described (24). All cross-links were cross-referenced using pLink (version 2.3.11) (25). Both Sim-XL and pLink files were searched against a custom FASTA file containing sequences for human APOA1, APOA2, APOE, APOM, serum amyloid A-1, and serum amyloid A-4 which were identified from proteomics analysis. APOE, APOM, serum amyloid A-1, and serum amyloid A-4 served as “dummy” control to set a scoring threshold for positive identifications and rule out false cross-links. A scoring threshold of 3.0 was used for Sim-XL resulting in only a single cross-link on the dummy proteins, while in pLink, threshold scores of 1.09 × 10^−3^ and 6.27 × 10^−1^ were used, at which dummy proteins showed no cross-linked peptide for BS3-H_12_ and BS3-D_12_, respectively. The assigned cross-links identified by SIM-XL and pLink were subjected to manual validation using XCalibur (version 4.5.474). Peptides cross-linked with DSBU were analyzed using Scout (26), with DSBU selected as the MS-cleavable crosslinker and FDR set to 1%. A noncrosslinked control sample was included to ensure robust crosslink assignments. Lastly, peptides cross-linked with DSSO were analyzed using a combination of Proteome Discoverer (v2.2) and XlinkX (v2.0). DSSO crosslinks were determined using a minimum XlinkX scoring threshold of 100 and a 1% FDR as determined using Percolator (v3.0, University of Washington). All cross-links included in the current studies had to be identified in at least two independent cross-linking experiments or two independent searches.

### Site-directed mutagenesis

For expressing all the APOA1 mutant proteins, the pET-30 APOA1 (UniProt ID: P02647) plasmid was used as a template to construct mutant plasmids by site-directed mutagenesis (27). In the mutant APOA1 (Δ1-43), the first 43 amino acid from the APOA1 protein were removed by proteolytic cleavage using TEV protease. This was accomplished by site-directed mutagenesis following Agilent protocol (Catalog no. 200518), by adding a TEV protease cleavage site to cleave after amino acid N43. The APOA1 (Δ220-243) mutant, where the last 24 amino acids are removed was constructed by adding a STOP codon after amino acid L219. Both truncation mutants were generated using the mature human APOA1 cDNA in a pET-30 vector. The dAPOA1^C-N^ mutant was equivalent to two APOA1 proteins connected tail-to-head with three alanine residues as linkers. This was accomplished by inserting a second copy of the APOA1 coding sequence immediately after the first with T4 DNA ligase (New England Biolabs), per the manufacturer's instructions, terminating with a stop codon. Then, site-directed mutagenesis (Agilent) was used to add three alanines in between the two copies of APOA1. The constructs were expressed in cells, purified by the integrated 6x His tag followed by proteolytic removal of the N-terminal tag as described previously (15). The APOA1 mutant proteins were used to synthesize reconstituted HDL particles with POPC as described above. Addition of APOA2 was performed at a 1:1 M ratio with the mutant APOA1 for the cholesterol efflux assays.

### Cholesterol efflux assay of plasma and PEG-depleted plasma with J774 cells

Cholesterol efflux via ABCA1 was tested in whole human plasma and APOB-depleted plasma using PEG (MW 6000, Sigma Aldrich; St. Louis, MO). J774 cells were grown to 80% confluency in DMEM/high glucose media plus 5% FBS and 1% penicillin/streptomycin at 37°C in a humidified 5% CO_2_ atmosphere. Efflux was assessed as net movement of [^3^H] cholesterol from J774 macrophage cells in basal media (consisting of DMEM containing 0.2% BSA) with 2 μg/ml ACAT inhibitor (28). The macrophages were incubated with medium containing 0.3 mM 8-Br-cAMP and 1 μCi/ml [H^3^] cholesterol (PerkinElmer Life Sciences) for 16 h in 5% CO_2_ at 37°C, washed twice with sterile PBS, and then incubated for 6 h with treatments (10 μg/ml protein) in media containing cAMP. Medium alone and medium containing 10 μg/ml of lipid-free APOA1 isolated from plasma were included as controls. Cholesterol efflux was measured by harvesting the medium, passing it through a 0.45 μM filter, and quantifying radiolabeled cholesterol in 100 μl of sample by liquid scintillation counting. Total cell counts were determined by extracting intracellular cholesterol from cells using isopropanol, drying under air, solubilizing in toluene, and counting. The percent efflux was calculated by dividing the number of counts in the medium by the total internalized counts per well (determined from the medium-only-treated cells). Percent efflux was normalized to the (−) acceptor controls.

### Cholesterol efflux assay of HDL and rHDL with BHK cells

Cholesterol efflux via ABCA1 was tested in APOA2-enriched UC-HDL and rHDL particles. In this assay, BHK cells that can toggle ABCA1 expression on or off using a mifepristone-inducible promoter were used. BHK cells were cultured in DMEM/high glucose media plus 5% FBS and 1% penicillin/streptomycin at 37°C in a humidified 5% CO2 atmosphere. Cells were treated with or without 10 nM mifepristone (Invitrogen Cat#H110-01) and all cells were radiolabeled by 1 μCi/ml [H3] cholesterol (PerkinElmer Life Sciences) for 16 h, washed twice with sterile PBS, and then incubated for 6 h with treatments (10 μg/ml protein) ± mifepristone. Medium alone and medium containing 10 μg/ml of lipid-free APOA1 isolated from plasma were included as controls. Cholesterol efflux was measured by harvesting the medium, passing it through a 0.45 μM filter, and quantifying radiolabeled cholesterol in 100 μl of sample by liquid scintillation counting. Total cell counts were determined by extracting intracellular cholesterol from cells using isopropanol, drying under air, solubilizing in toluene, and counting. The percent efflux was calculated by dividing the number of counts in the medium by the total internalized counts per well (determined from the medium-only-treated cells). Percent efflux was normalized to the (−) acceptor controls.

## Results

### Exogenous APOA2 enhances cholesterol efflux to human plasma

In previous work, we observed that intact human plasma HDL particles containing both APOA1 and APOA2 (LpA-I/A-II) promoted more efficient cholesterol efflux via ABCA1 than did HDL particles lacking APOA2 (LpA-I) (13). This was surprising because the general dogma holds that lipidated HDL particles participate minimally in ABCA1-mediated cholesterol efflux with the lion’s share mediated by lipid-poor APOA1 (29). We first set out to investigate if this observation was simply an artifact of studying isolated lipoprotein particles. We reasoned that if the APOA2 effect were important, we should be able to demonstrate an increase in ABCA1-mediated cholesterol efflux when exogenous lipid-free APOA2 is added directly to human plasma. We note that lipophilic molecules like APOA2 and APOA1 likely do not exist as completely "lipid-free" in human plasma; here, they serve as models for "lipid-poor" molecules, which lack sufficient lipid to form HDL-like discs or spherical particles.

**Fig. 1:**
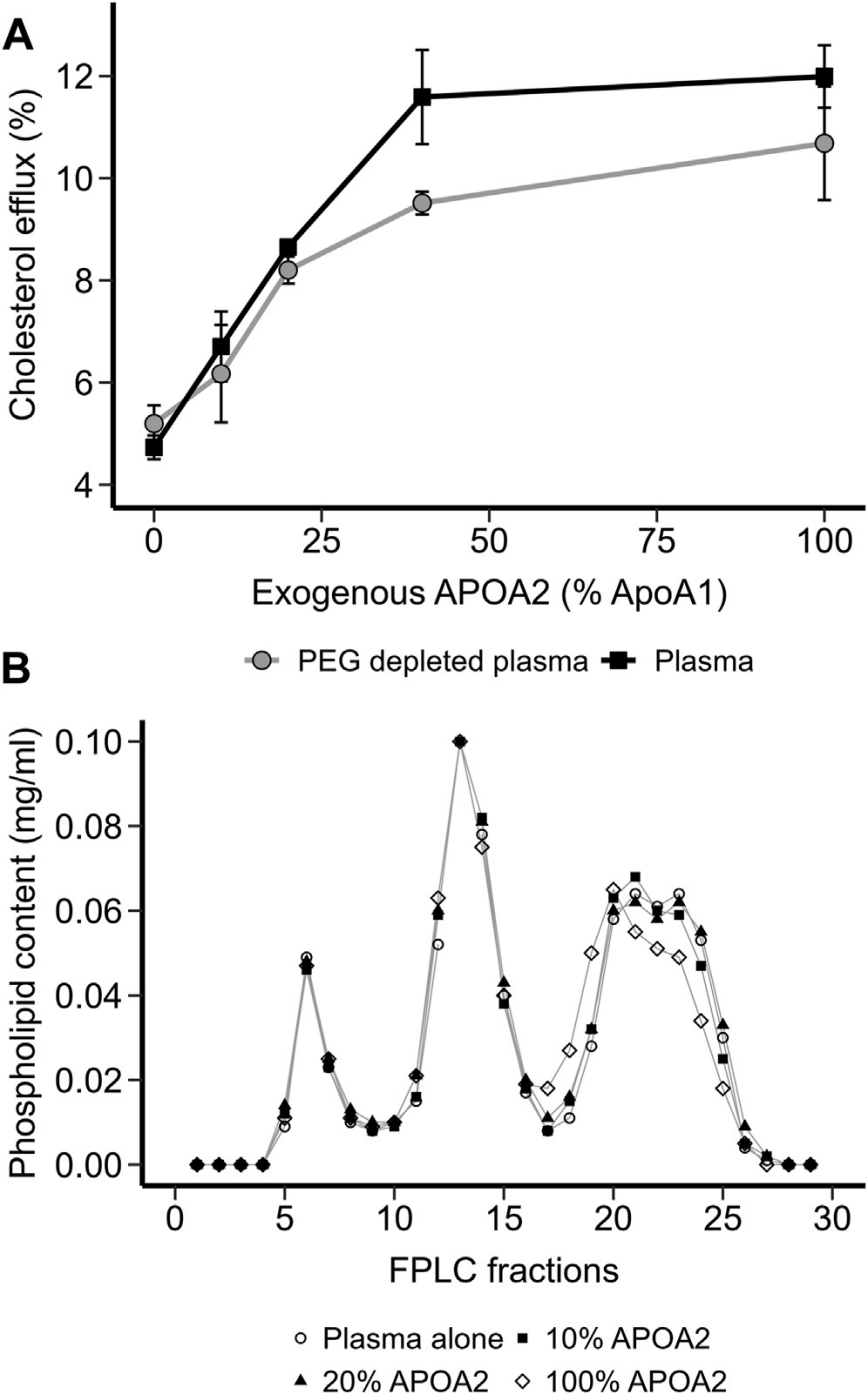
Effect of APOA2 addition on cholesterol efflux and lipoprotein distribution in human plasma. A: ABCA1-mediated cholesterol efflux from J774 cells to either 2% human plasma or 2% human plasma with APOB-containing lipoproteins depleted by PEG precipitation. Plasma and PEG-depleted plasma samples were incubated with increasing amounts of exogenous APOA2, before the cholesterol efflux assay wherein ABCA1 expression was induced by cAMP. Data points and error bars represent the mean and SD from three technical replicates, respectively. B: phospholipid profile of human plasma incubated with increasing amounts of APOA2 from panel A. Plasma was incubated with purified human APOA2 for 1 h at 37°C and then applied to tandem Superose 6 size-exclusion columns. Each fraction was analyzed for choline-containing phospholipids using a colorimetric assay.

Plasma was obtained from a normolipidemic human volunteer with no indication of cardiovascular disease. The plasma sample was split into two aliquots with one treated with PEG to precipitate APOB-containing lipoproteins (see *Methods*) and the other treated with buffer alone. Purified human APOA2 was incubated directly with plasma or the HDL-containing supernatant from the APOB-depleted half for 1 h at 37°C. The APOA1 concentration in plasma was found to be ∼1.5 mg/ml by Clinical analyzer (Randox Rx). APOA2 was added at 0 (buffer only), 10%, 20%, 40%, and 100% of APOA1 on a mass basis. This corresponded to final APOA2 concentrations of 0, 0.15, 0.3, and 1.5 mg/ml, respectively. Each aliquot was diluted to 2% (v/v) in cell culture medium and used as acceptors for ABCA1-mediated cholesterol efflux in J774 cells (see *Methods*). Figure 1A shows that both native and APOB-depleted plasma promoted the release of ∼ 5% of cellular cholesterol without additional APOA2. As the amount of exogenous APOA2 was progressively increased to about 40% of total APOA1 plasma content, cholesterol efflux increased almost linearly. This happened regardless of the presence of APOB-containing lipoproteins, suggesting they played a minor role in the efflux enhancement. No additional improvement came with APOA2 beyond 40%. Figure 1B shows the same plasma samples analyzed on a double Superose 6 size-exclusion system. Tracking the lipoproteins by phospholipid content, the VLDL and LDL particle profiles were not altered by APOA2 at any concentration. The HDL peak was also minimally affected by the 10% and 20% additions, but changes in size distribution were apparent at 100% APOA2 addition. Thus, the increase in cholesterol efflux due to APOA2 addition was effectively related to changes in the HDL population alone. Since APOA2 circulates in human plasma at concentrations between 0.25 and 0.40 mg/ml (8), the most potent efflux enhancement effects were within the physiological range.

### Exogenous APOA2 enhances ABCA1-mediated cholesterol efflux to lipidated HDL particles

**Fig. 2:**
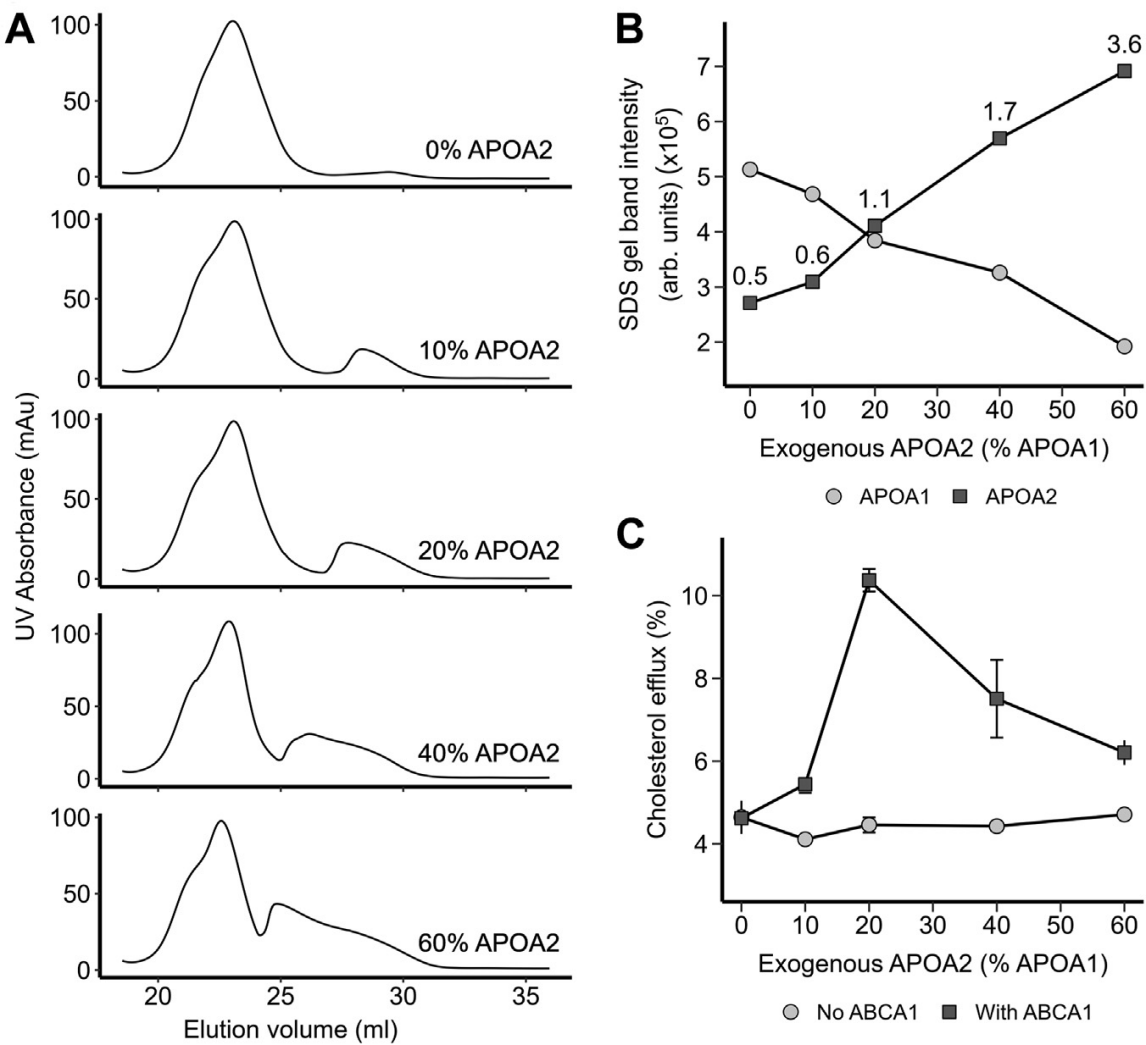
Effect of APOA2 on ABCA1-mediated cholesterol efflux to isolated HDL. A: size-exclusion profiles of UC-isolated HDL incubated with increasing concentration of exogenous APOA2 for 1 h and immediately separated on tandem Superdex 200 columns. Peaks eluting between 20-25 ml and 25–30 ml were HDL-bound (lipidated) and displaced (lipid-free) protein fractions, respectively. B: SDS-PAGE-based densitometry analysis of HDL-bound fractions shows the change in APOA1 and APOA2 content with increasing addition of exogenous APOA2. The APOA2:APOA1 band intensity ratios are indicated above each APOA2 data point. C: cholesterol efflux ability of HDL-bound fractions to mediate cholesterol efflux in BHK cells with and without ABCA1 expression. Data points and error bars represent the mean and SDs from three technical replicates, respectively.

APOA2 is well known to displace APOA1 from HDL particles (30, 31, 32, 33). Therefore, a trivial explanation for the results in Figure 1 is that the addition of APOA2 displaced APOA1 from HDL particles, generating lipid-poor forms, known to be potent acceptors of ABCA1-mobilized cholesterol. To test this, we incubated ultracentrifugally (UC) isolated total HDL with lipid-free APOA2 at 0% (only buffer added), 10%, 20%, 40%, and 60% of HDL APOA1 content. After the incubation, the HDL particles were isolated away from any displaced APOA1 by SEC and then tested on cells with and without ABCA1 expression (See supplemental Fig. 1 for the experimental workflow). Figure 2A shows the ultraviolet light absorbance (280 nm) profile of the size-based separations after APOA2 incubation. HDL showed a wide profile from 19 to 26 ml consistent with the known size diversity of HDL. As APOA2 was added, a peak appeared to the right (ie, smaller size). SDS PAGE analysis showed that this new peak was primarily composed of APOA1 and that the majority of added APOA2 appeared in lipidated HDL particles (supplemental Fig. 2), consistent with its known high lipid affinity. The liberated APOA1 peak increased in area (ie, amount) as well as molecular size as more APOA2 was incubated with the HDL. The former was likely because APOA2 displaced increasing amounts of APOA1 from the HDL particles. The latter may be due to APOA1’s tendency to self-associate in its lipid-poor form in a concentration-dependent manner. The HDL particles eluting between 19-24 ml were pooled to isolate them from lipid-poor apolipoproteins. Figure 2B shows the results of an SDS-PAGE densitometry analysis that tracked the abundance of APOA1 and APOA2 in the isolated HDL samples. As expected, APOA1 was the dominant band in the baseline HDL samples. As APOA2 was increasingly added, the amount of APOA1 remaining in the particles decreased steadily, while the amount of APOA2 residing in the particles increased. At the highest end of the APOA2 dose, about 70% of APOA1 had been displaced from the particles in favor of APOA2.

The isolated APOA2-enriched HDL particles from Figure 2A were tested for their ability to accept cholesterol using the BHK cell system that contains a mifepristone-inducible cassette to induce ABCA1 expression (34). This system has advantages over the cAMP inducible system used in Figure 1 in that it specifically targets ABCA1 expression whereas the cAMP used in the macrophages has myriad additional effects on the cells (35). Supplemental Fig. 3 shows the control samples included in the cholesterol efflux assays performed in this study. It is clear that ABCA1 expression promoted robust cholesterol efflux to lipid-free APOA1 and to a lesser extent to lipid-free APOA2, but ABCA1 expression did not significantly affect cholesterol efflux to fully lipidated reconstituted HDL particles containing APOA1 and with synthetic POPC. Figure 2C shows the ability of the isolated APOA2-enriched HDL particles to promote cholesterol efflux in this system. With no added APOA2, the HDL particles promoted efflux of about 4% of cell cholesterol. In the absence of ABCA1, this value did not change with the additions of APOA2. However, in the presence of ABCA1, cholesterol efflux increased with APOA2 addition, peaking at 20% and then returning to nearly baseline at the highest levels of APOA2 addition. Comparing Figures 2A–C, maximal ABCA1-mediated cholesterol efflux was promoted by the HDL particles when the intensity of the APOA1 and APOA2 bands were about equal. After that, ABCA1-mediated cholesterol efflux declined as APOA1 levels dropped. Since these particles were isolated away from any liberated lipid-poor APOA1, these results do not support the hypothesis that APOA2 enhances cholesterol efflux solely through the generation of lipid-poor apolipoprotein species.

**Fig. 3:**
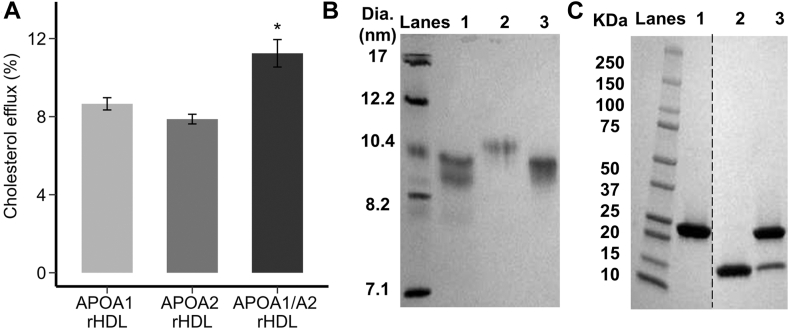
Ability of different scaffold proteins in reconstituted HDL particles to promote ABCA1-mediated cholesterol efflux. A: cholesterol efflux from BHK cells with ABCA1 expression for reconstituted HDL (rHDL) containing APOA1 alone, APOA2 alone, and APOA1/APOA2 showing that both APOA1 and APOA2 are required for enhanced ABCA1-mediated cholesterol efflux. Particles were compared at equal phospholipid concentration. B: native PAGE analysis of reconstituted HDL particles produced with POPC. Lanes 1, 2, and 3 show reconstituted HDL particles containing APOA1, APOA2, and APOA1/APOA2, respectively. C: SDS-PAGE analysis (nonreducing) of the rHDL particles showing APOA1 (28 kDa) and/or APOA2 (14 kDa). Lanes 1, 2, and 3 show reconstituted HDL particles containing APOA1, APOA2, and APOA1/APOA2 particles. All gels were loaded with equal protein and stained with Coomassie blue. Bars and error bars represent the mean and SDs from three technical replicates, respectively. Star (∗) represents a difference from the APOA2-only rHDL particle by Kruskal-Wallis and Dunn’s post-hoc test at *P* < 0.05.

The results of the previous experiment suggest that APOA2 itself is not the sole factor that is responsible for the cholesterol efflux enhancement effect. The incubations at higher ratios of APOA2 generated HDL particles that were dominated by APOA2, yet cholesterol efflux to those particles was declining. To more conclusively verify that APOA2 itself was not the operational factor driving high cholesterol efflux, we generated rHDL particles with synthetic POPC that contained APOA1 alone, APOA2 alone, or a combination of both. Figure 3A shows that ABCA1-mediated cholesterol efflux was not different between particles containing solely APOA1 or APOA2. However, consistent with the experiments above using isolated human HDL, the presence of both APOA1 and APOA2 on the rHDL particles resulted in enhanced ABCA1-mediated cholesterol efflux. Native PAGE analysis of the rHDL particles showed that the hydrodynamic diameters of the particles were roughly similar (Fig. 3B) and an SDS PAGE analysis confirmed their protein composition (Fig. 3C). This shows that APOA2 itself has no inherent advantage over APOA1 and both proteins were required together on HDL particles to enhance ABCA1-mediated cholesterol efflux.

### APOA2 alters APOA1 structure on HDL particles

Since APOA1 is the primary protein responsible for interaction with ABCA1, albeit in its lipid-poor form, we hypothesized that APOA2 may alter the conformation of HDL-bound APOA1 to mimic the ABCA1-interacting properties of lipid-free APOA1. To test this, we used limited proteolysis, a bottom-up proteomics-based method to interrogate how APOA2 affects APOA1 structure on HDL (20, 21). We performed a similar experiment to the one shown in Figure 2. Increasing amounts of APOA2 (0%, 20%, 40%, 80%, and 160% of total APOA1 content) were titrated into UC-isolated HDL and incubated at 37°C for 1 h. As shown in Figure 2A, following incubation, the HDL-bound proteins and the proteins displaced due to APOA2 addition were isolated by SEC and pooled separately. The HDL-bound proteins underwent limited proteolysis, while the liberated proteins were only subjected to the traditional bottom-up proteomics workflow (supplemental Fig. 1).

Briefly, the HDL-bound proteins were digested with nonspecific protease, Proteinase K for 1 min. Then the particles were denatured, and all proteins were exhaustively digested with trypsin. This double-digestion results in a mixture of semitryptic and tryptic peptides. The changes in semitryptic peptides outline structural changes, as they are produced by the action of Proteinase K on the nondenatured protein. An identical set of HDL-bound proteins was subjected to tryptic digestion only and used to control for APOA1 abundance changes under each condition. Conversely, the displaced protein fractions were denatured with urea and digested with trypsin (supplemental Fig. 1). The resultant mixtures of digested peptides were analyzed using tandem mass spectrometry and label-free quantification to identify APOA2-induced abundance and structural changes. Supplemental Fig. 4 shows the changes in the relative abundance of APOA1 and APOA2 in the HDL-bound (left) and liberated lipid-poor proteins (right). Consistent with Supplemental Fig. 2, label-free quantification shows that the increasing binding of APOA2 to HDL particles leads to proportional displacement of APOA1 molecules as shown by the almost exponential decay in APOA1 levels (supplemental Fig. 4). At the highest level of exogenous APOA2, there is a 95% drop in the APOA1 level in the HDL-bound fraction compared to the control group where no exogenous APOA2 was added. This is further confirmed by a gradual accumulation of APOA1 in the liberated protein fractions. Additionally, the increasing APOA2 levels in the liberated fractions with increasing exogenous APOA2 can be attributed to the excess APOA2 that fails to bind to the HDL particles during the 1h incubation. It should be noted that following complete digestion, the displaced/free protein fractions from the control groups (0% exogenous APOA2) did not yield sufficient peptide quantity for mass spectrometry analysis.

**Fig. 4:**
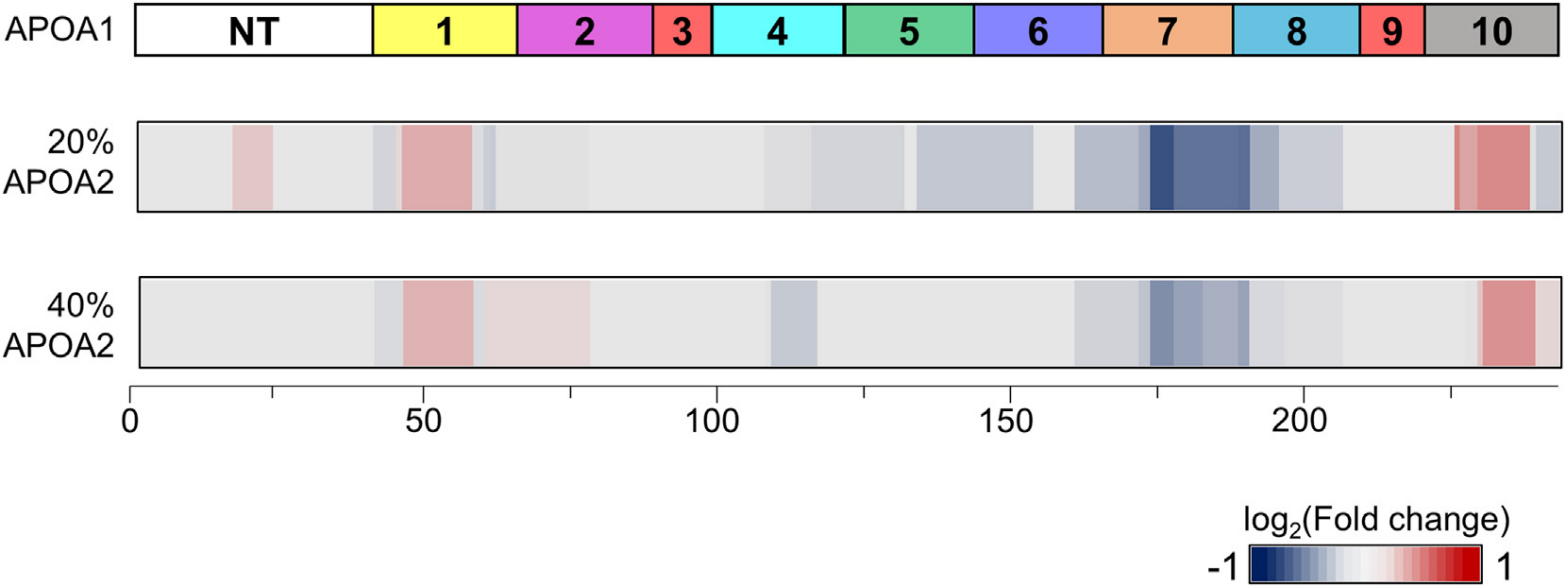
Structural changes in APOA1 in the presence of exogenous APOA2. Top bar shows cartoon with the 10 amphipathic alpha-helical repeats as reported by *Segrest et al*. as a reference for the structural barcodes. Residue-level structural changes in APOA1 in the presence of 20% (*middle*) and 40% (*bottom*) exogenous APOA2 are represented as 2-D structural barcodes of APOA1. The log2 fold changes of significant semi-tryptic peptides from five biological replicates (*P*-value < 0.01) are distributed across the encompassing residues. Regions in *red* get more exposed to the nonspecific protease and the regions shown in the *blue* are shielded from the nonspecific protease upon the exogenous addition of APOA2.

The accompanying structural changes induced by gradual APOA2 titration are visualized as structural barcodes (Fig. 4 and supplemental Fig. 5), which show the change in solvent accessibility of different APOA1 regions with added APOA2 (see Methods). The barcodes show the residue-level fold changes between APOA2 treatment and control of statistically significant semitryptic peptides (*P*-value <0.01). Regions indicated in shades of red have higher relative abundance in the experimental groups than the negative control, suggesting preferential digestion by Proteinase K under nondenaturing conditions and have higher solvent accessibility in the presence of exogenous APOA2. Conversely, blue regions indicate lower abundance, implying reduced accessibility compared to the control. To judge the authenticity of the limited proteolysis results, HDL was supplied with a reliable negative control (carbonic anhydrase at 65% of APOA1 content), which led to the identification of minimal structural changes as expected (supplementary Fig. 5). However, drastic structural changes were observed at multiple APOA1 regions in the presence of exogenous APOA2. At both 20% and 40% APOA2, a decrease in solvent accessibility is observed around helix H7, indicating APOA2 interacts with APOA1 in this region (Fig. 4). Conversely, there is an increase in solvent accessibility at the N terminus, H1, and H10, indicating these regions become more exposed in the presence of exogenous APOA2. At higher levels of exogenous APOA2 (80% and 160%), additional structural changes occur albeit without a clear pattern (supplemental Fig. 5). It should be noted that at these conditions, most APOA1 is driven off the particles by APOA2 (supplemental Fig. 4), which might explain the aberrant patterns of solvent accessibility. Taken together, these data show that APOA2 can alter APOA1 conformation in intact HDL particles, particularly in the C- and N-terminal regions that have been associated with ABCA1 interactions, at least in lipid-poor APOA1 (see Discussion). When compared with Figure 2C, the highest cholesterol efflux is observed at 20% APOA2, with modest efflux still observed at 40% APOA2. This led us to hypothesize that the enhanced cholesterol efflux in the presence of APOA2 is driven by the higher accessibility and in turn, more interaction of APOA1 N- and C-terminus with ABCA1.

### Identification of site(s) of interaction between APOA1 and APOA2 in HDL by chemical cross-linking

To further explore the physical interaction between APOA2 and APOA1 in HDL, we used chemical cross-linking combined with mass spectrometry to identify sites of proximity in the native HDL particle structure. For rigor, and to reduce the possibility of false positives, we utilized three separate crosslinking agents: BS^3^-H_12_/D_12_ (homobifunctional for Lys with isotope tagging), DSBU (homobifunctional for Lys, with MS cleavage), and DSSO (homobifunctional for Lys, with MS cleavage). UC-isolated HDL was treated with various concentrations of BS^3^-H_12_/D_12_ and the extent of cross-linking was monitored by SDS-PAGE (reducing conditions) after quenching the reaction (data not shown). Untreated HDL showed two major bands near 25 kDa and 8 KDa, which correspond to APOA1 and a reduced monomer of APOA2, respectively. After cross-linking with BS^3^-H_12_/D_12_ at 50:1 M ratio of BS^3^:APOA1, these bands were significantly reduced in favor of new, diffuse bands at higher MW (supplemental Fig. 6). After delipidation and tryptic digestion, cross-linked peptides were identified by mass spectrometry, and intermolecular cross-links were confirmed by MS/MS spectra showing fragment ions from both APOA1 and APOA2 (see *Methods*). We confidently identified and manually validated eight intermolecular cross-links (Table 1). Similar experiments were performed with DSBU and DSSO and cross-links were verified by the presence of signature ions upon dissociation in MS2 (see *Methods*). We identified nine intermolecular linkages with both reagents (Table 2). Three cross-links (APOA1 – APOA2); Lys59 – Lys30, Lys182 – Lys39, Lys182 – Lys46, were found with all three reagents and are visualized in supplemental Fig. 7. In general, the cross-links were concentrated at the ends of the APOA1 in helix 1 at the N terminus and helices 7, 8, and 10 at the C terminus. No interactions were noted between helix 2 and the middle of helix 7. In general, these data are consistent with APOA2-induced changes in proteolytic exposure (supplemental Fig. 7).

**Table 1:** Identified BS3 cross-links between APOA1 and APOA2

Residue^a^		Peptide		Parameters		
APOA1	APOA2	APOA1	APOA2	Mass of H_12_ (Da)^b^	Mass of D_12_ (Da)^c^	PPM
K59	K30	LLDNWDSVTSTFSKLR	VKSPELQAEAK	3220.7	3232.8	2.453
K182	K30	LEALKENGGAR	VKSPELQAEAK	2497.4	2509.5	5.384
K182	S40	LEALKENGGAR	SPELQAEAKSYFEK	2925.5	2937.6	0.016
K182	K46	LEALKENGGAR	SKEQLTPLIK^e^K	2750.7	2762.7	2.359
K195	K46	LAEYHAKATEHLSTLSEK	SKEQLTPLIK	3325.8	3337.9	3.620
K208	K30/S31/K39	AKPALEDLR	VKSPELQAEAK^d^SYFEK	3162.7^f^	3174.8^f^	2.812^f^

a Cross-linker was BS3 H_12_/D_12_, a homobifunctional and isotopically coded crosslinker capable of joining primary amino groups.

b Experimentally measured total mass of BS3 H_12_ crosslinked peptides.

c Experimentally measured total mass of BS3 D_12_ crosslinked peptides.

d Mono-link of BS3 H_12_ (156.0786 Da).

e Mono-link of BS3 D_12_ (168.1528 Da).

f The cross-linking of APOA1 K208 – APOA2 K30.

**Table 2:** Identified DSBU/DSSO cross-links between APOA1 and APOA2

Residue^a^		Peptide		Parameters			
APOA1	APOA2	APOA1	APOA2	Mass (Da)		PPM	
				DSBU	DSSO	DSBU	DSSO
K40	K30	DSGRDYVSQFEGSALGKQLNLK	VKSPELQAEAK	3810.01	3771.91	2.160	1.876
K59	K30	LLDNWDSVTSTFSKLR	VKSPELQAEAK	3279.73	3241.67	0.837	0.834
K182	K28	LEALKENGGAR	DLMEKVK	2234.20	2196.11	3.988	0.975
K182	K39	LEALKENGGAR	SPELQAEAKSYFEK	2982.53	2944.45	1.435	1.257
K182	K46	LEALKENGGAR	SKEQLTPLIK(K^b^)	2512.43	2603.45	1.033	1.885
K208	K30	AKPALEDLR	VKSPELQAEAK	2409.34	2372.26	1.243	1.664
K208	K39	AKPALEDLR	SPELQAEAKSYFEK	2837.49	2799.40	2.167	1.518
K208	K44	AKPALEDLR	SYFEKSK	2098.13	2061.05	3.749	1.571
K226	K30	QGLLPVLESFKVSFLSALEEYTK	VKSPELQAEAK	3996.19	3958.09	2.465	1.938

a Cross-linker was BS3 H_12_/D_12_, a homobifunctional and isotopically coded crosslinker capable of joining primary amino groups.

b SKEQLTPLIKK is peptide for DSSO.

### The C terminus of APOA1 is required for the APOA2 cholesterol efflux enhancement effect

**Fig. 5:**
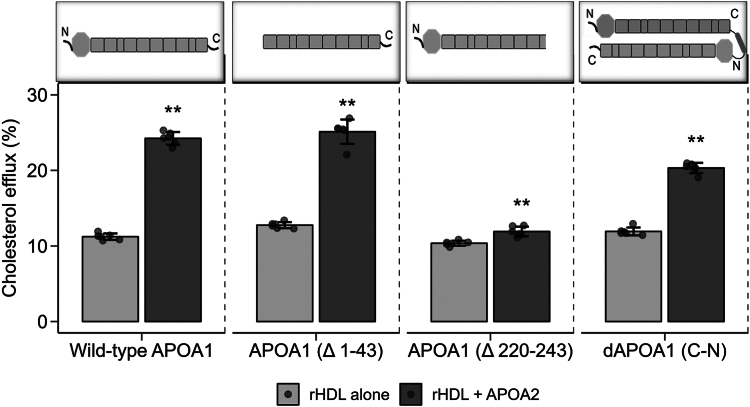
Cholesterol efflux to rHDL particles produced with various mutants of APOA1. Cholesterol efflux from BHK cells expressing ABCA1 is shown. All the particles were reconstituted with POPC at an approximate wt:wt ratio of 2.5 mg POPC/1.0 mg WT APOA1 or mutant. *Gray* bars show the rHDL particles containing the WT APOA1 or mutant indicated alone. *Black* bars show the same particle with added APOA2 (at about 20% of APOA1 mass). Data and error bars represent the mean and SDs from six technical replicates. The data from individual replicates is shown along each bar. ∗∗*P* < 0.01 by Mann-Whitney test compared to corresponding non-APOA2 containing rHDL particle.

The data presented above supported the hypothesis that exogenous APOA2 affects the conformation of native APOA1 on HDL particles. To confirm if these conformation changes are responsible for the enhanced cholesterol efflux, additional cholesterol efflux assays were performed with reconstituted HDL-containing APOA1 mutants. Since the proteolytic sensitivity and the cross-linking implicated the N- and C-termini of APOA1, we generated rHDL particles with recombinant APOA1 deletion mutants that lack *i*) the N-terminal 43 a.a. (APOA1^Δ1-43^) and the C-terminal 23 a.a. (APOA1^Δ220-243^) (supplemental Fig. 8). These complexes were then incubated with and without APOA2 and tested for ability to promote ABCA1-mediated cholesterol efflux in the BHK cell system. Figure 5 shows that APOA2 addition to WT APOA1 enhanced cholesterol efflux beyond the APOA1-containing particles alone as expected. Similarly, addition of APOA1 to rHDL particles containing APOA1^Δ1-43^ also stimulated additional cholesterol efflux. By contrast, addition of APOA2 to rHDL particles that contained APOA1^Δ220-243^ failed to increase cholesterol efflux. This is even though all APOA1 mutants were able to produce rHDL particles of reasonably similar size to WT APOA1 and each mediated roughly similar cholesterol efflux prior to the addition of APOA2 (data not shown). To confirm that the effects were not due to liberation of APOA1 from the particles, we performed a similar experiment with a covalent dimer of APOA1 that was created by linking the C terminus of one APOA1 to the N terminus of another (dAPOA1^C-N^) (36). Being covalently attached, it was unlikely that added APOA2 could entirely displace the APOA1 dimer from the rHDL particle. The covalently dimerized APOA1 also showed an enhancement of cholesterol efflux though not as extensive as WT APOA1. Although there are technically two APOA1 molecules wrapped around the discoidal particle in the dAPOA1^C-N^ containing particle, only one of them has a free C terminus, which may explain the reduced effect of APOA2 addition in this case. Overall, these experiments indicate that the APOA2 enhancement of ABCA1-mediated cholesterol efflux to preformed particles containing APOA1 requires at least one free C terminus on the resident APOA1 particles.

## Discussion

The key findings in this study are as follows: *i*) human APOA2 boosts the ability of lipidated HDL particles to accept cholesterol efflux from cellular ABCA1, *ii*) this occurs at concentrations that reflect the range of APOA2 concentrations in human plasma, *iii*) the APOA2 effect is only observed in the presence of APOA1 on the same particles, *iv*) APOA2 can alter the structure of APOA1 at its termini, and *v*) an intact APOA1 C-terminus is required for the APOA2 enhancement effect. The implications of these results to our understanding of the mechanisms of cholesterol efflux to HDL are discussed below.

Lipid-poor apolipoproteins are well-documented to promote cholesterol removal from cells via a mechanism that is distinct from their fully lipidated counterparts (37). Indeed, once ABCA1 was shown to be the key cellular mediator of lipid efflux to lipid-poor apolipoproteins, the dogma has been that lipid-containing HDL particles accept cellular cholesterol via aqueous diffusion mechanisms mediated by SR-B1 and ABCG1 (see (6) for a good review) but do not interact significantly with ABCA1 (38). However, this idea has been called into question by the studies of Du *et al*. (39), which clearly showed that small HDL particles can promote cholesterol efflux via ABCA1 with some populations rivaling the efficiency of lipid-free APOA1. Following up, He *et al*. (40) proposed that APOA1’s C-terminal helical regions are unstably bound to small HDL particles and can disassociate from the particle surface. The implication is that these “flipped-ends” might then be free to interact with ABCA1 and promote cholesterol efflux in much the same way that lipid-poor APOA1 does. This notion is supported by recent models for the interaction of free APOA1 with ABCA1 by Segrest *et al*. (41), which hold that APOA1 may bind patches of cellular phospholipid that have been sequestered away from the cell surface by the pumping action of ABCA1. The mechanism by which small HDL particles may liberate APOA1 domains is not fully clear, but it may involve high particle surface curvature.

It would seem that these data require a revision of the dogma surrounding lipid-poor APOA1 and ABCA1. Rather than strict limitation to lipid-poor apolipoproteins, acceptors of ABCA1-mediated cholesterol efflux should be thought of as a continuum in which lipid-poor apolipoproteins make the most efficient acceptors (because they start out largely empty) but other small, but clearly lipid-containing particles, can also participate with decreasing effectiveness as they become more lipidated. This is important because clinical studies are beginning to show that extra small HDL particles are highly correlated to cholesterol efflux efficiency in diabetes (42, 43).

Our current report adds further support to the idea that some lipidated HDL particles participate in interactions with ABCA1. Although APOA2 clearly displaces APOA1 from HDL particle surfaces at high concentrations, we showed that the liberated APOA1 was not responsible for the overall effect of increased efflux to intact HDL nor was the effect directly due to APOA2 itself because *i*) the efflux effect diminishes under conditions where APOA2 becomes the dominant apolipoprotein in HDL and *ii*) rHDL particles composed entirely of APOA2 showed no inherent benefit over those generated entirely with APOA1. The efflux enhancement was only observed when the two proteins were present together in significant amounts suggesting these proteins are acting cooperatively. Indeed, limited proteolysis results revealed that the enrichment in APOA2 increases the solvent exposure of APOA1 C terminus, like the “flipped C-terminal ends” present on small HDL (40). Furthermore, functional studies with APOA1 mutants corroborated that APOA1 C terminus is required for APOA2 effect.

**Fig. 6:**
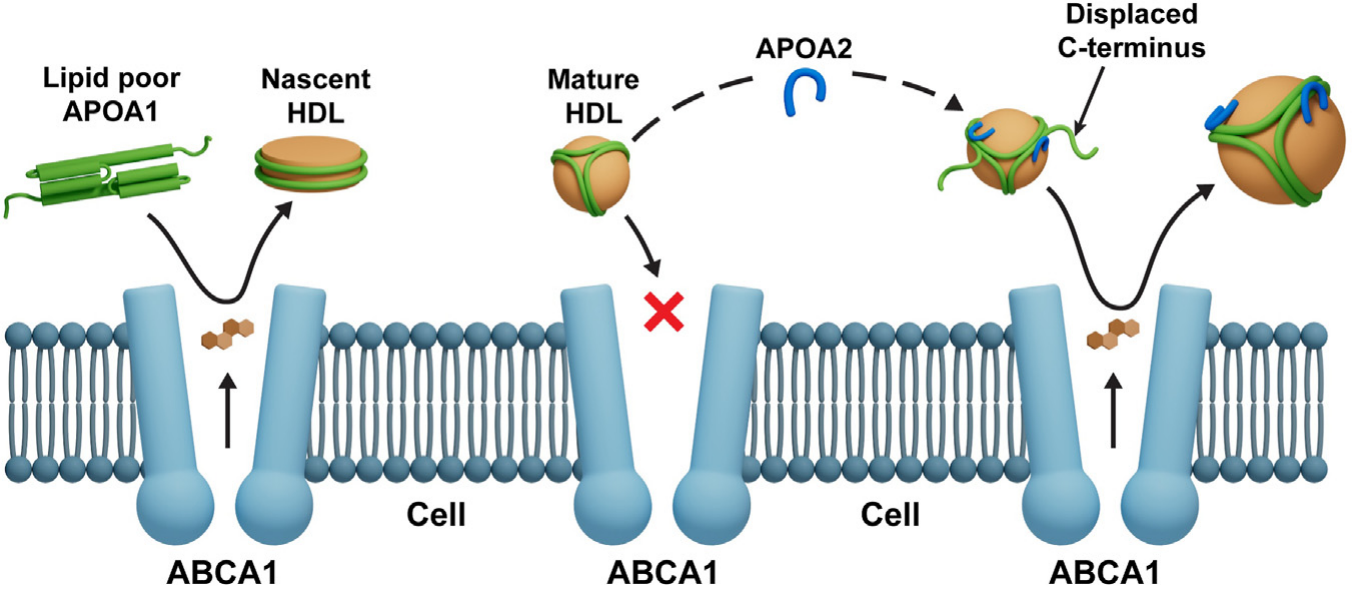
Cartoon showing mechanism of increase in ABCA-1-mediated cholesterol efflux induced by exogenous APOA2. Lipid-free APOA1 interacts with ABCA1 through its exposed C-termini and acts as an efficient acceptor of cholesterol. However, in mature HDL, the APOA1 termini are in close contact with the lipoprotein surface, hindering interaction in ABCA1. Driven by the structural and functional evidence at hand, we propose that APOA2 leads to the dislodgement of Helix 10 away from the lipid surface, making it available to form auxiliary interactions with ABCA1, thereby increasing the rate of apolipoprotein-mediated cholesterol efflux.

Given these data, our current model for the APOA2 effect mirrors the “flipped ends” hypothesis for small HDL. Figure 6 presents a cartoon of the APOA2-induced structural changes in HDL that increases its ability to accept cholesterol. In the lipid-poor state, the free C-termini of APOA1 effectively interacts with ABCA1 to mediate cholesterol efflux. However, in mature HDL, APOA1 is firmly attached to spherical HDL particles with both its termini in close contact with the surface lipids. We suggest that additional APOA2 binding causes the C-terminal helices of APOA1 to disengage from the particle surface, allowing them to interact extensively with ABCA1 similarly to small HDL particles as suggested by He *et al*. (40). Alternatively, or perhaps in addition, these freed ends may allow the particle to microsolubilize more cellular lipid from ABCA1-generated cell surface patches as proposed by Phillips et al. (44). The mechanism by which APOA2 orchestrates this structural change is not clear though our data indicates that APOA2 specifically alters the C-terminal domain of APOA1 (Fig. 4). The idea of partially displaced APOA1 has support from interfacial biophysical studies. For example, parts of APOA1 can be pushed off a triolean/water interface at lower surface pressures (∼15 nm/m) than is required to eject the full-length protein (∼19 mN/m) (45, 46). Furthermore, numerous laboratories have shown that the extreme C-terminal helical domains of APOA1 are critical for lipid-poor APOA1 to effectively accept cholesterol via ABCA1 (47, 48).

However, we note that our model is at odds with previous notions on the role of APOA1 domains in lipid binding and dissociation. The C-terminal helix 10 of APOA1 is one of the most surface active in the protein (49). In fact, helix 10 and helix 1 are likely the domains that first contact lipid. Once anchored, they have been proposed to trigger the unfolding of the soluble APOA1 helix bundle to extend into the lipid-bound belts characterizing lipid-bound APOA1. Furthermore, peptide mimetics of helix 10 exhibit one of the highest surface exclusion pressures compared to those of other helical domains in APOA1 (49). Wang *et al*. proposed a model for APOA1 desorption from lipid surfaces in which the APOA1 N-terminal residues desorb first, followed by the central domains with the C-terminal residue leaving last (50). In this context, our proposed APOA2-induced dissociation of the APOA1 C terminus is surprising. However, most APOA1 desorption studies use an experimentally induced increase in lipid packing density (using a surface balance or an oil -drop tensiometer) to drive it off the interface. This suggests that APOA2 may not eject APOA1 by increasing lipid surface pressures past the exclusion point of certain APOA1 domains. How it accomplishes this is not clear, but our cross-linking data suggests that specific protein:protein contacts between APOA2 and APOA1 are responsible for the altered lipid surface association properties of APOA1. For this reason, it is important to derive a detailed understanding of APOA1/APOA2 structural interactions in HDL, a major future goal of our laboratories.

### A physiological role for APOA2 in cholesterol efflux?

Prior to about 2000, numerous studies reported conflicting conclusions as to the effect of APOA2 on cholesterol from cultured cells. These experiments were performed in widely differing cell types including hepatocytes (51), adipocytes (52), fibroblasts, and smooth muscle cells (53) and focused primarily on the aqueous diffusion modes of cholesterol efflux. Once the role of ABCA1 as the primary mediator of cAMP-induced efflux was understood (54), Fournier showed that plasma from transgenic mice overexpressing human APOA2 was more efficient than control plasma in removing macrophage cholesterol when ABCA1 expression was stimulated by cAMP (55). This fits with our studies of human LpA-I and LpA-I/A-II particles with similar results (13). On the other hand, a study from Rotllan et al. showed that chow-fed APOA2-transgenic mice exhibited similar levels of macrophage-specific reverse cholesterol transport as the control (56). This may indicate that human APOA2 introduced into the mouse system may have complex effects across the various steps of the RCT pathway. Interestingly, HDL isolated from transgenic rabbits expressing APOA2 exhibited increased CEC versus WT control (57). Furthermore, the rabbits developed less atherosclerosis despite similar cholesterol levels as controls (57). Rabbit studies may have more relevant insights into the human lipoprotein biology than mouse studies because they utilize APOB-containing lipoproteins for cholesterol transport and have CETP activity (58). As for human clinical studies, Koekemoer *et al*. performed a large-scale population study (nearly 2000 individuals) to identify molecular determinants of cholesterol efflux. Efflux was positively associated with HDL levels as expected, but it should be noted that APOA2 was found to be significantly associated with high CEC (59). Overall, the previous literature and our current findings suggest that APOA2 should not be ignored when contemplating therapeutic strategies for improving cholesterol efflux. Specifically, the ability of APOA2, in the presence of APOA1, to increase ABCA1-mediated efflux to fully lipidated HDL particles could be therapeutically harnessed. Although the efflux to these particles may not be as efficient as that observed from an equal amount of lipid-free APOA1, when one considers that the lion’s share of circulating APOA1 resides in these particles (<95%, vs. >5% as lipid-poor forms), this may represent a high-capacity therapeutic target. It may be possible to design therapies that not only increase cholesterol efflux to lipid-poor acceptors but also to preformed HDL particles.

## A historical note

When originally characterizing the ability of ABCA1 to promote cholesterol efflux to extracellular acceptors, Oram *et al*. (54) beautifully showed that ABCA1 expression is stimulated by cAMP in macrophages and that cholesterol and phospholipid efflux proceeds remarkably efficiently to lipid-free proteins like APOA1. In that same paper, they found that cAMP also stimulated cholesterol efflux to isolated human HDL particles (quite substantially, see Fig. 1A of (54)), but not to HDL that had been subjected to trypsinization. The interpretation was that HDL preparations contain some amounts of contaminant lipid-free apolipoproteins that account for the increased efflux in the presence of ABCA1. This observation was likely an origin of the oft-repeated statement that ABCA1 only works with lipid-free or lipid-poor apolipoprotein acceptors. We have run hundreds of cholesterol efflux experiments in our lab over the last two decades and almost always include UC-isolated HDL as a control. We commonly see that induction of ABCA1 results in more cholesterol efflux to total HDL. This does not occur when we use only APOA1-containing rHDL particles (supplemental Fig. 3). We suggest that the differences in efflux between HDL with and without ABCA1 induction may be due, at least in part, to a contribution of spherical LpA-I/A-II particles that can interact with ABCA1.

## Data Availability

Primary liquid chromatography-mass spectrometry (LC-MS) raw measurement data reported in this study are openly accessible for download at the Mass Spectrometry Interactive Virtual Environment (MassIVE) community repository under the following accession URI: https://identifiers.org/massive:MSV000095479 and can be formally cited using the MassIVE dataset doi: https://doi.org/10.25345/C59W0998W. Additional processed data files and supporting materials are openly accessible at the Predictive Phenomics Initiative (PPI) Project data catalog for download, under the "Profiling the Structural Proteome to Define Phenotype" project data package doi: https://doi.org/10.25584/PPI/2447854. Project DataHub download (∼2.8 GB) includes sample naming key, processed MaxQuant (v1.6.17.0) results, and resulting protein annotated relative abundance files. Linked accessions and relevant computational source code package RomicsProcessor (https://github.com/PNNL-Comp-Mass-Spec/RomicsProcessor) supporting data transparency and reuse can be found at the DataHub doi download page.

## Supplemental data

This article contains supplemental data.

## Conflicts of Interests

The authors declare that they have no conflicts of interests with the contents of this article.
