# Supplementary material for: APOA2 increases cholesterol efflux capacity to plasma HDL by displacing the C-terminus of resident APOA1
Source: J Lipid Res. 2024 Oct 28;65(12):100686. doi: 10.1016/j.jlr.2024.100686 (PMC11617996; doi:10.1016/j.jlr.2024.100686)
Supplement: Supplemental Data [file mmc1.docx]

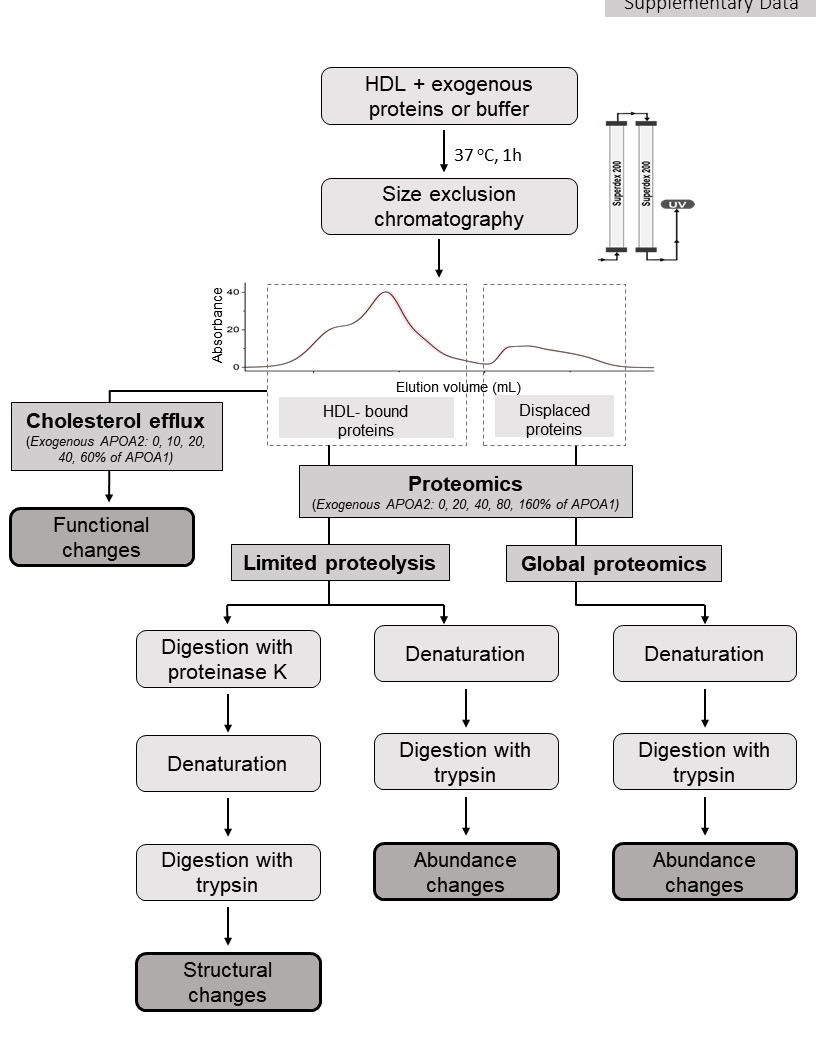
**Supplementary figures**

**Supplementary Figure 1.** Schematic representation of the cholesterol efflux and limited proteolysis experimental workflow. HDL isolated from human plasma was treated with exogenous proteins (APOA2 or carbonic anhydrase) or buffer and passed through two size exclusion columns. The remodeled HDL-bound proteins and displaced proteins were collected and pooled separately. The HDL- bound fractions were subjected to a cholesterol efflux assay or limited proteolysis while the displaced lipid-free proteins were only analyzed using putative bottom-up proteomics. For limited proteolysis, the proteins were incubated with nonspecific protease, Proteinase K for a short duration, followed by denaturation and complete tryptic digestion. This workflow resulted in proteolytic fingerprints that informed us about structural changes within the HDL structure. The bottom-up proteomics workflow involved denaturation with urea and treatment with trypsin alone, which revealed information about changes in protein abundances.


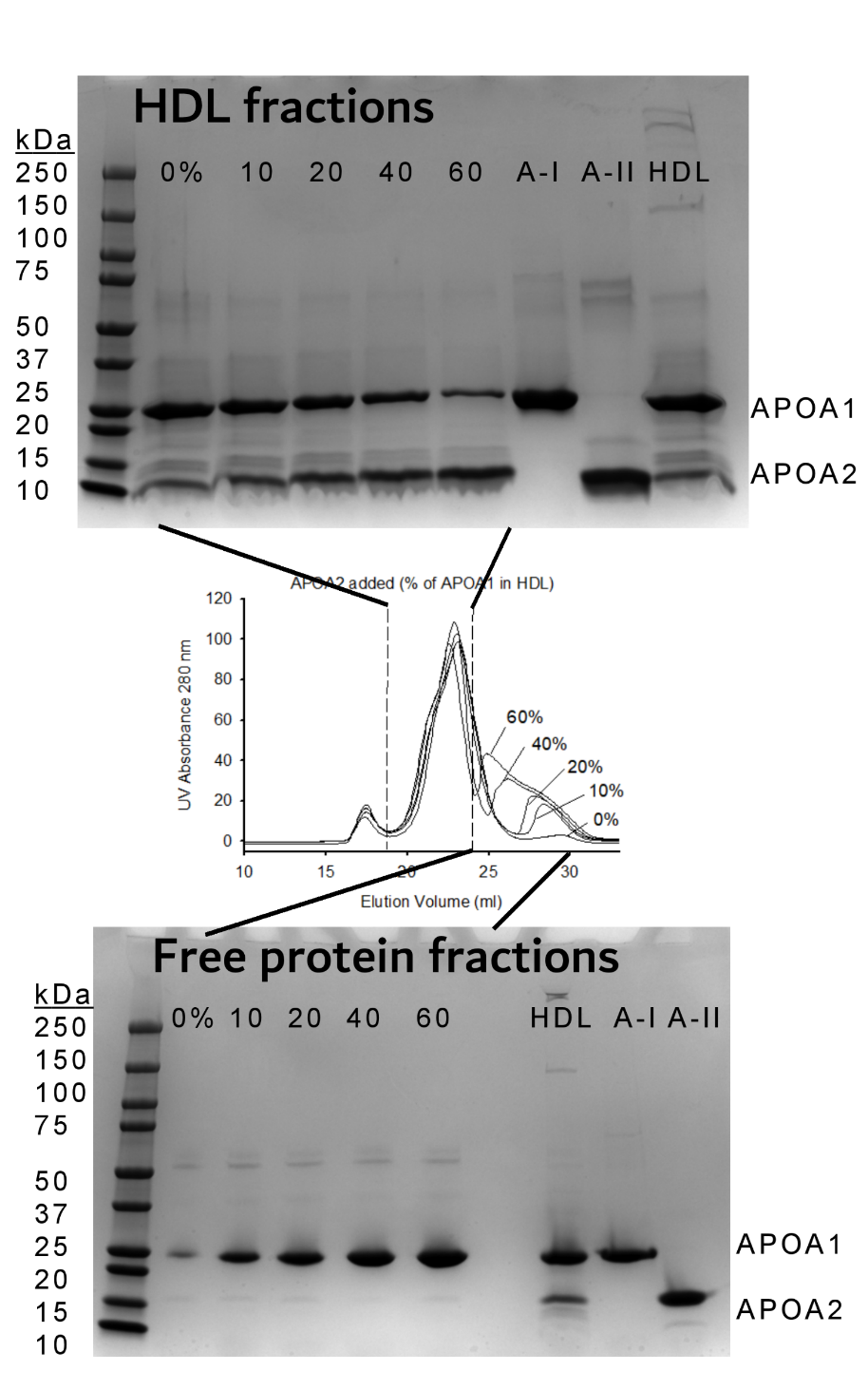


**Supplementary Figure 2. SDS-PAGE analysis of human plasma HDL incubated with various amounts of purified APOA2.** The size exclusion chromatography traces in the center are those from **Fig. 2a**. **Top)** SDS-PAGE of pooled HDL fractions (between dotted lines). This gel was analyzed by densitometry to produce **Fig. 2b**. Note the reduction of APOA1 remaining with the HDL particles after the column separation and the increase in HDL-associated APOA2 as more APOA2 was added to the incubation. Bottom panel shows the SDS-PAGE of pooled “free” protein fractions after combining, dialyzing into ammonium bicarbonate buffer, then concentrating. Note that APOA1 increasingly appears in this zone as more APOA2 was incubated with HDL. On both gels, purified APOA1 (A-I) and APOA2 (A-II) are shown for reference along with the HDL sample prior to any treatment. All gels stained with Coomassie blue.


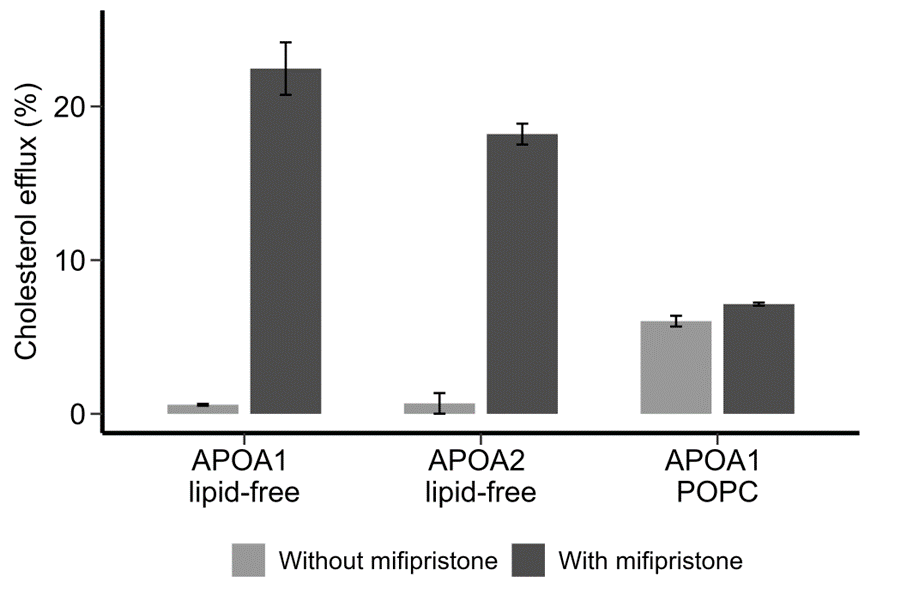


**Supplementary Figure 3. Typical ABCA1-mediated cholesterol efflux control samples using the BHK inducible ABCA1 system.** Cholesterol efflux from BHK cells with (dark gray) and without ABCA1 expression (light gray). ABCA1 expression was induced by incubation with mifipristone. In every experiment, we run 10 µg/ml of lipid-free APOA1 to verify that cholesterol efflux increases dramatically upon ABCA1 induction. The same mass of lipid-free APOA2 also induces ABCA1-mediated cholesterol efflux, though not to the extent of APOA1. However, a recombinant HDL (APOA1 POPC) particle composed of APOA1 (20 µg/ml of phospholipid) and synthetic POPC (dia ~96 Å) induces cholesterol efflux similarly irrespective of ABCA1 expression. This is thought to be due to diffusional mechanisms mediated by SR-BI or ABCG1. This shows that the BHK cell system cleanly distinguishes between acceptors that favor the two general cholesterol efflux mechanisms. Bars and error bars represent the mean and standard deviations from 3 technical replicates, respectively.


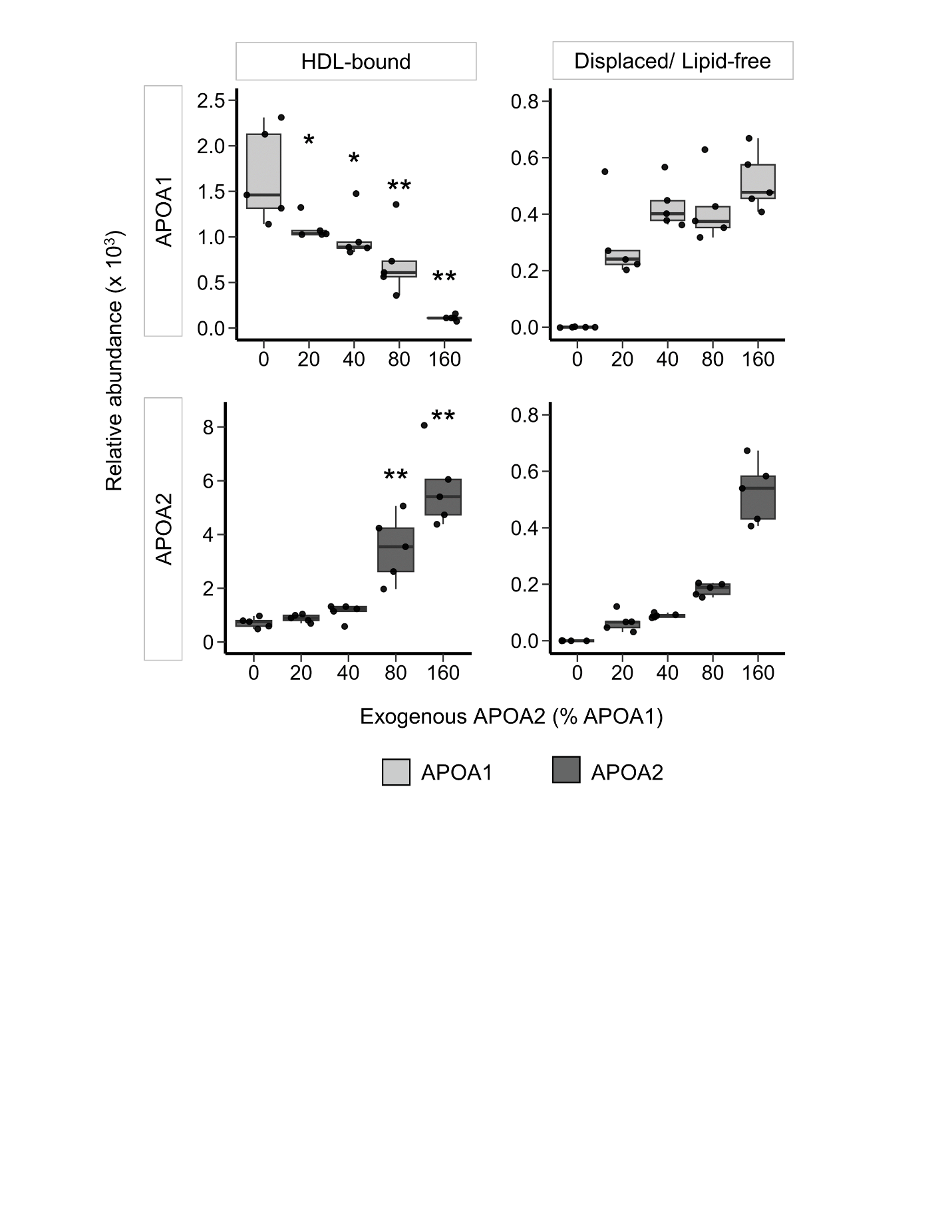


**Supplementary Figure 4**. **Mass spectrometry-based quantification of HDL-bound and free protein fractions.** The relative abundance of APOA1 and APOA2 is measured using untargeted proteomics of HDL-bound (left panel) and displaced free protein fractions (right panel) obtained by titrating increasing amounts of APOA2 into UC-isolated HDL. The relative abundances are expressed as normalized label-free quantification (LFQ) intensities. Data represents the median and whiskers represent the range of 5 biological replicates. Data points from each replicate are also represented along each box. One asterisk (*) represents p-value <0.05 and two asterisks (**) represents p<0.01 for a student t-test comparing HDL samples with and without exogenous APOA2. The shown analytes were also found to be significantly different across the five groups by the Kruskal-Wallis test.


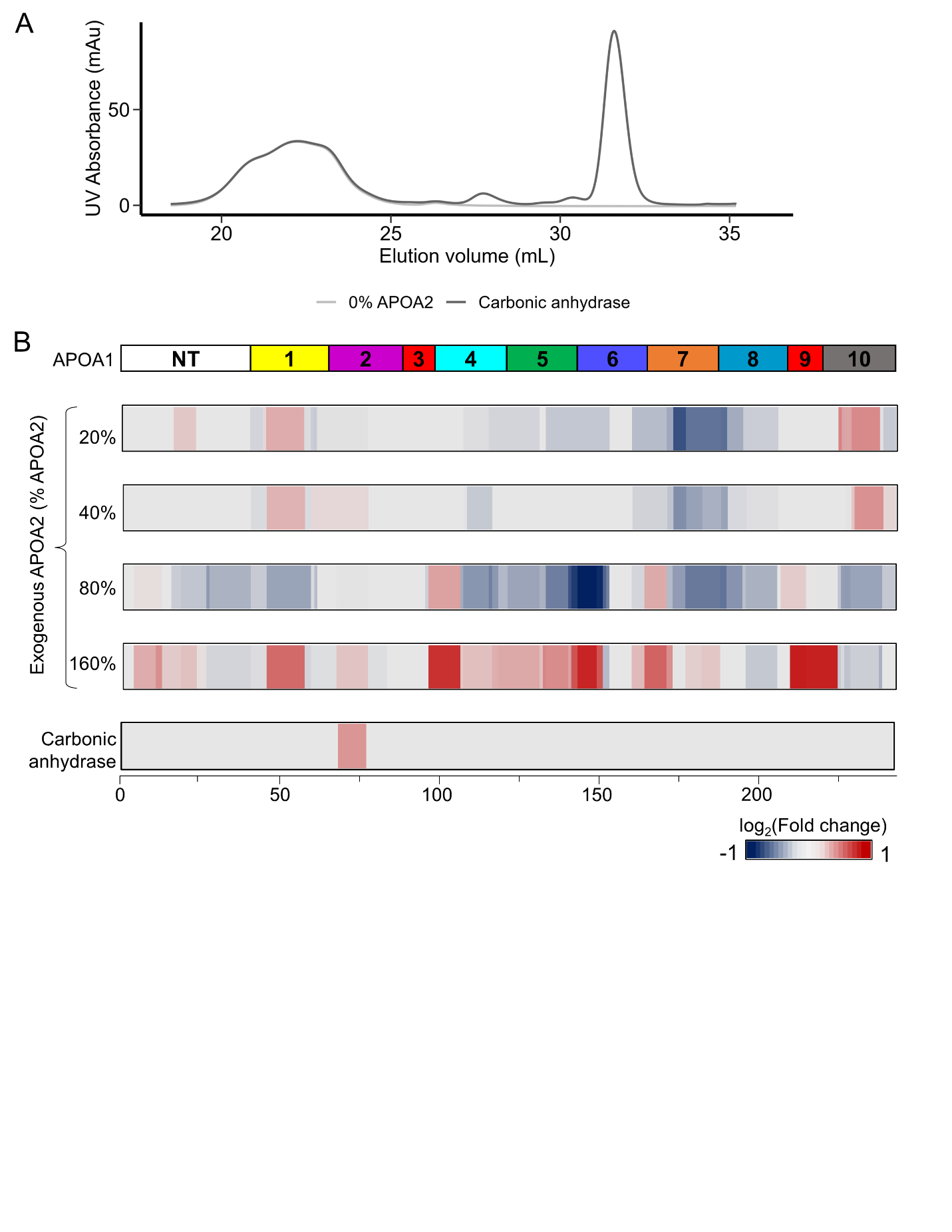


**Supplementary Figure 5. Structural changes in APOA1 in the presence of exogenous APOA2 or carbonic anhydrase.** A) As a negative control, carbonic anhydrase was incubated with ultracentrifuged HDL for 1h, and immediately separated over tandem Superdex 200 columns. The resultant size exclusion profile shows no significant changes in the HDL-bound protein fractions (20-25 ml) in the presence of carbonic anhydrase making it an ideal negative control. B) Top bar shows cartoon with the 10 amphipathic alpha-helical repeats as reported by *Sergest et. al* as a reference for the structural barcodes*.* Residue-level structural changes in APOA1 in the presence of 20%, 40%, 80% and 160% exogenous APOA2 or exogenous carbonic anhydrase (negative control) are represented as 2-D structural barcodes of APOA1. The log2 fold changes of significant semi-tryptic peptides from 5 biological replicates (p-value < 0.01) are distributed across the encompassing residues to obtain the 2D-barcodes. Regions in red get more exposed to the non-specific protease and the regions shown in the blue are shielded from the non-specific protease upon the addition of exogenous proteins. Note that carbonic anhydrase induces negligible changes to APOA1 structure as indicated by the lack of significant semi-tryptic peptides.


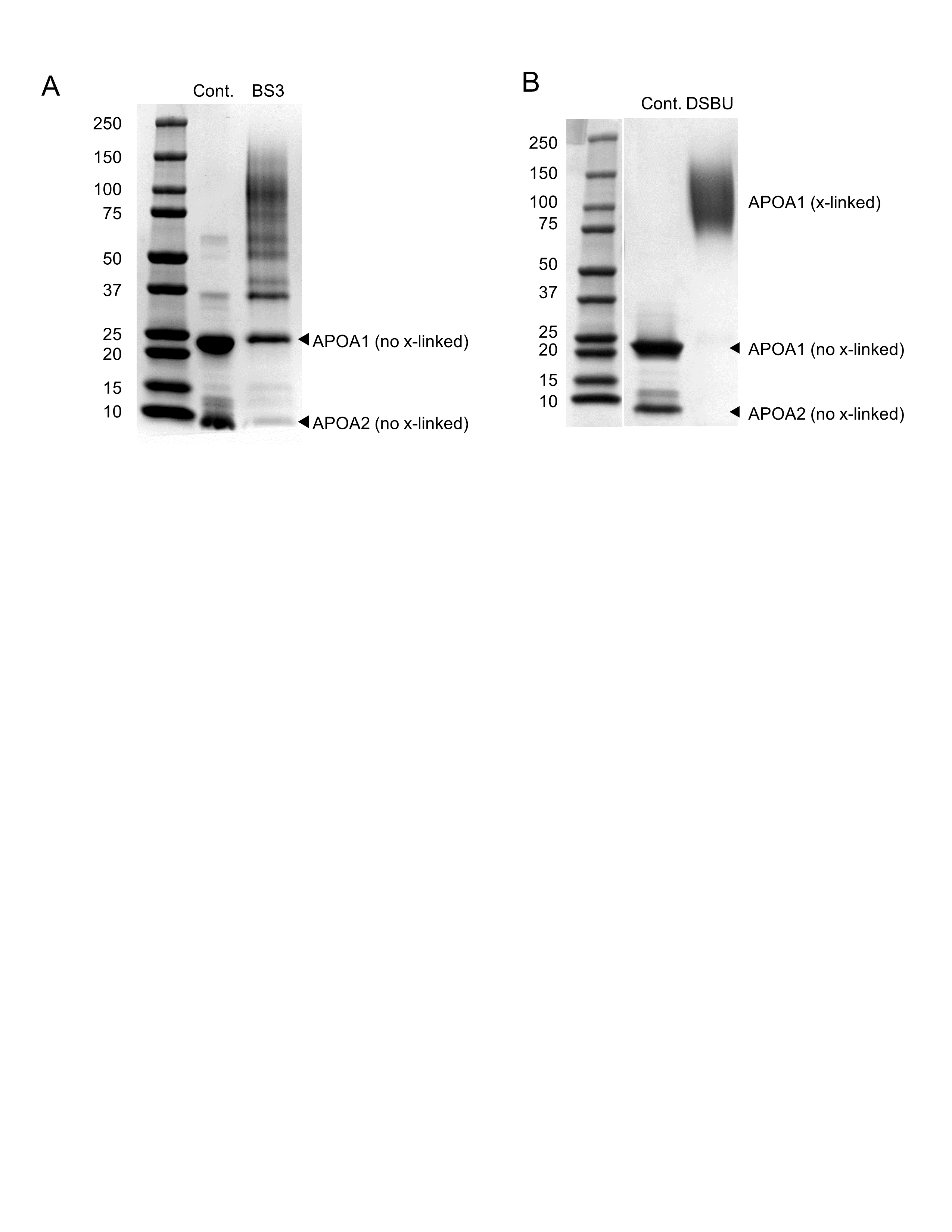


**Supplementary Figure 6. Cross-linked APOA1 and APOA2 through BS3-H_12_/D_12_or DSBU.** Proteins in UC- isolated HDL of human plasma were cross-linked through BS3-H_12_/D_12_ (A), and DSBU (B). Arrows indicate the bands supposed to APOA2 (9.303 kDa) and APOA1 (28.961 kDa).


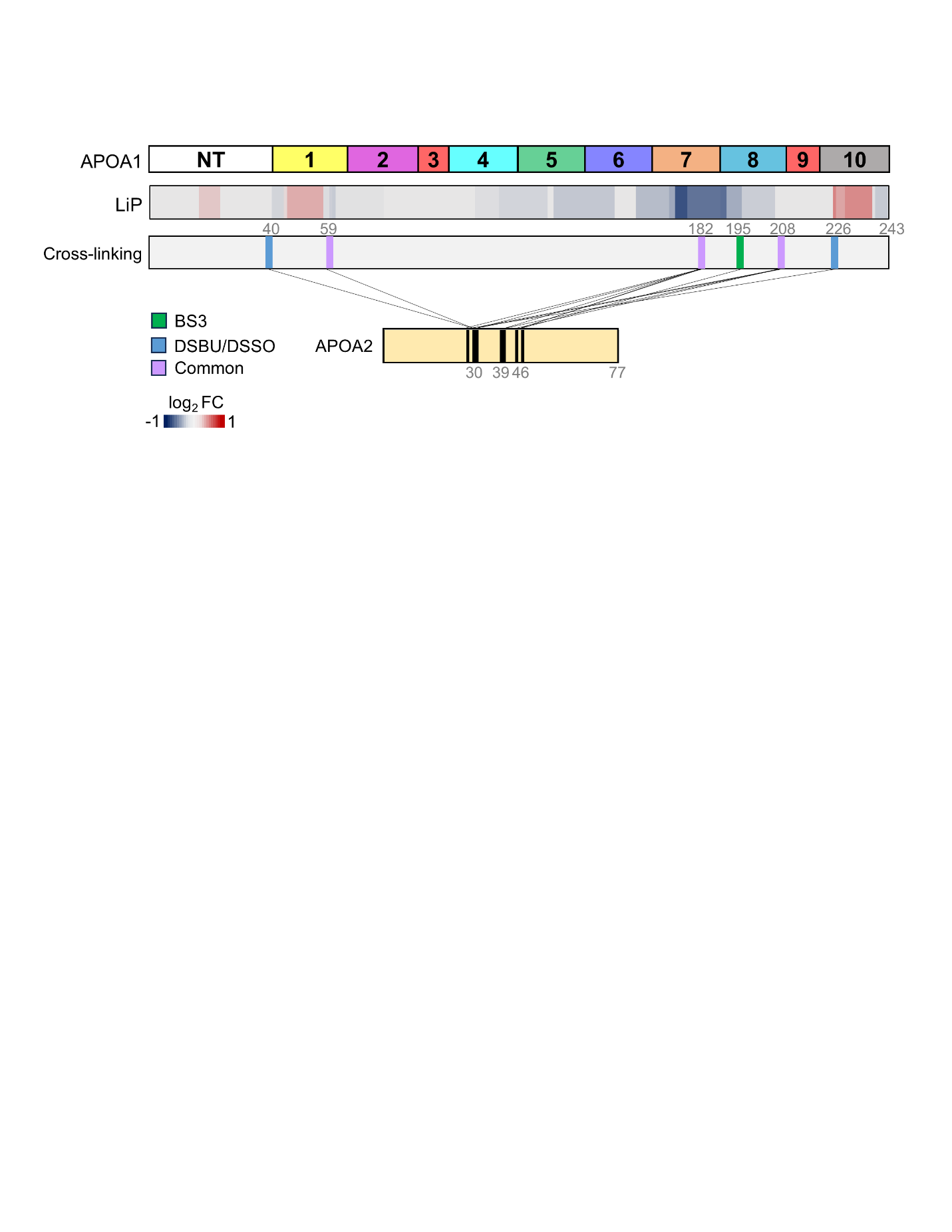


**Supplementary Figure 7.** **LIP and cross-linking data comparison.** A graphical summary of LiP (at 20% APOA2) and cross-linking (with BS3 and DSBU/DSSO) results show the identified peptide regions and amino acid sites affected by APOA2.


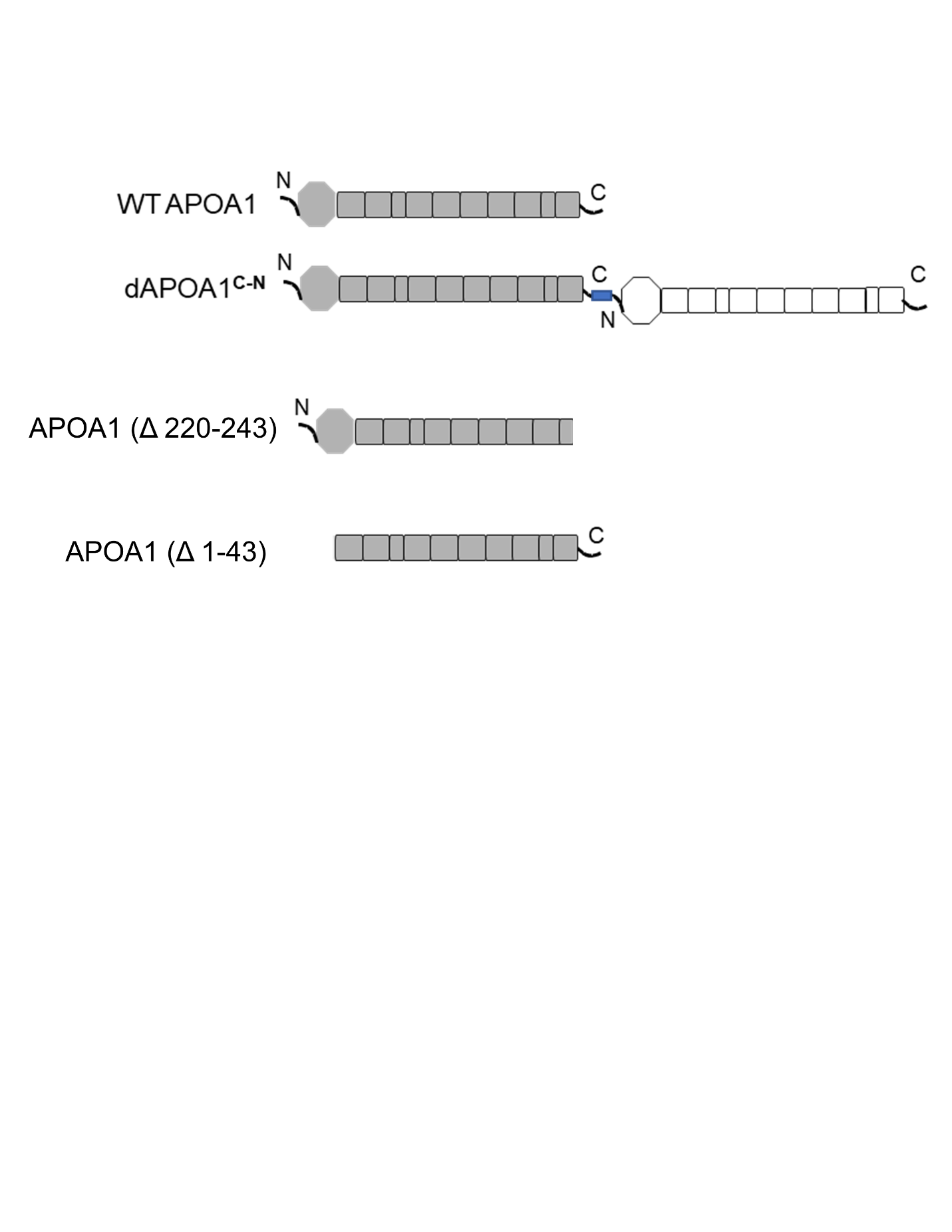


**Supplementary Figure 8.** Engineered mutants of APOA1 to identify domain responsible for APOA2 enhancement of cholesterol efflux.
